# A Review on Binderless Tungsten Carbide: Development and Application

**DOI:** 10.1007/s40820-019-0346-1

**Published:** 2019-12-30

**Authors:** Jialin Sun, Jun Zhao, Zhifu Huang, Ke Yan, Xuehui Shen, Jiandong Xing, Yimin Gao, Yongxin Jian, Hejie Yang, Bo Li

**Affiliations:** 1 State Key Laboratory for Mechanical Behavior of Materials, School of Materials Science and Engineering, Xi’an Jiaotong University, Xi’an, 710049 People’s Republic of China; 2 Key Laboratory of High Efficiency and Clean Mechanical Manufacture of MOE, School of Mechanical Engineering, Shandong University, Jinan, 250061 People’s Republic of China; 3 Key Laboratory of Education Ministry for Modern Design and Rotor-Bearing System, Xi’an Jiaotong University, Xi’an, 710049 People’s Republic of China; 4 School of Mechanical and Automotive Engineering, Qilu University of Technology, Jinan, 250353 People’s Republic of China

**Keywords:** Binderless tungsten carbide, Sintering, Densification, Toughening, Mechanical properties

## Abstract

WC-Co alloys have enjoyed great practical significance owing to their excellent properties during the past decades. Despite the advantages, however, recently there have been concerns about the challenges associated with the use of Co, i.e. price instability, toxicity and properties degeneration, which necessitates the fabrication of binderless tungsten carbide (BTC). On the other hand, BTC or BTC composites, none of them, to date has been commercialized and produced on an industrial scale, but only used to a limited extent for specialized applications, such as mechanical seals undergoing high burthen as well as high temperature electrical contacts. There are two challenges in developing BTC: fully densifying the sintered body together with achieving a high toughness. Thus, this review applies towards comprehensively summarize the current knowledge of sintering behavior, microstructure, and mechanical properties of BTC, highlighting the densification improving strategies as well as toughening methods, so as to provide reference for those who would like to enhance the performance of BTC with better reliability advancing them to further wide applications and prepare the material in a way that is environment friendly, harmless to human health and low in production cost. This paper shows that the fabrication of highly dense and high-performance BTC is economically and technically feasible. The properties of BTC can be tailored by judiciously selecting the chemical composition coupled with taking into careful account the effects of processing techniques and parameters.

## Highlights

- Establish processing-composition-microstructure-property relationships governing binderless tungsten carbide (BTC).
- Highlight the densification improving strategies and toughening methods for BTC.
- Provide key challenges as well as the outlook for superior peformance associated with BTC.

## Graphical abstract

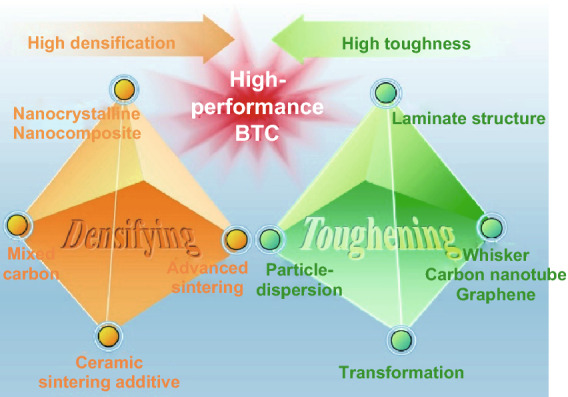

## Introduction

Cemented carbides enjoy great practical significance and have been extensively applied as rock drill buttons, pressing dies, cutting tools, and other abrasion-resistant engineering parts due to their combinations of considerable toughness, strength, and wear resistance [1–5]. The tungsten carbide phase endows the alloy excellent hardness as well as superior wear resistance, while the binder phase is responsible for strength and toughness of the composite materials.

Cobalt (Co) is the mostly employed binder phase during the conventional sintering process due to a combination of fundamental synergistic reasons including its favorable wettability on tungsten carbide, interfacial bonding strength between WC and Co, the plasticity of Co phase in the composite, and its own strength and wear resistance [6, 7]. However, its usage yields the deteriorations in hardness and oxidation/corrosion resistance along with elevated temperature performance owing to the inferior chemical characteristics of cobalt to the carbide phase. Furthermore, thermal stress is ease of generation because of the thermal expansion coefficients misfit [8–10]. When machining metal with high plasticity as pure iron using conventional WC–Co cemented carbide, chip tends to adhere on the rake face of cutting tool, resulting in serious adhesive wear due to the existence of cobalt with low melting point. This issue is exacerbated when cobalt resources may face depletion and economic crises for cobalt occurred because of the excessive development for growing application of cobalt for rechargeable accumulators (batteries) and in superalloys for high-performance jet turbines as illustrated in Table 1 [11]. Additionally, the acute pulmonary toxicity including on macrophage toxicity of WC and Co mixture has been proved to be higher than that of single WC and Co, making sintered WC–Co cemented carbides classified as carcinogenic to humans by International Agency for Research on Cancer (IARC) [12, 13].

**Table 1:** Overview of different uses of cobalt [11]

Product group	Form	Fraction of supply (%)	Trend in use and since when	Recycling	Value added to product
Super-alloys	Metallic	22	Increases 1995	Yes	High
Cutting tools	Metallic	11	Increases 1960	Partly	Medium
Magnets	Metallic	12	Increases 1990	Partly	High
Other applications	Metallic	11	Constant -	No	Low
Batteries	Chemical	22	Increases 2000	Yes	Medium
Chemicals, catalyst	Chemical	9	Increases 1960	No	Low
Resins, pigments	Chemical	13	Constant -	No	Low

**Fig. 1:**
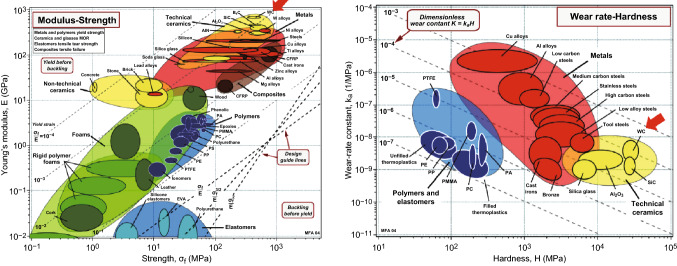
Ashby diagrams for materials. Left: Young’s modulus versus strength, right: wear-rate constant versus hardness. The red arrows point to tungsten carbides [15]. Figure panels reproduced from Ref. [15] with permission from Michael F. Ashby copyright 2009. (Color figure online)

On account of the reason above, considerable studies have paid overwhelming attention to the development of BTC. As pioneered by Kanemitsu [14] in 1982, BTC, also named binderless cemented carbide, is tungsten carbide free of any metallic binder including cobalt, iron, and nickel. As shown in Fig. 1, because of the absence of metal binder, BTC composites, possessing attractive properties as exceptional hardness as well as excellent wear/corrosion/oxidation resistance, can be fascinating materials serving in extremely tough environments. However, while initially appealing, none of them, to date, has been commercialized and produced on an industrial or commercial scale. BTCs are only used to a limited extent for specialized applications, such as mechanical seals undergoing high burthen, optical glass lens molds, nozzles for abrasive water jet as well as high-temperature electrical contacts. The production of high-performance BTC is even more challenging. The bottleneck lies in the difficulties encountered in consolidating and toughening BTC. More precisely, it is difficult to consolidate pure tungsten carbide to a full density through conventional sintering, due to its strong energy covalent chemical bonds and high melting point together with low self-diffusion coefficients. Furthermore, WC abnormal grain growth and the formation of sub-carbide W_2_C are also unavoidable. As a consequence, BTC composites usually exhibit such disadvantages such as unsatisfactory flexural strength and fracture toughness. In the backdrop of this problem, the development of BTC demands material design solving the twin problems of densification and brittleness.

The advanced sintering techniques, such as spark plasma sintering (SPS), reactive spark plasma sintering (RSPS), high-frequency induction-heated sintering (HFIHS) as well as pulsed current active sintering (PCAS), are successfully employed in laboratory scale preparing dense BTC. Particularly, SPS is currently the most applied sintering methods to consolidate BTC or BTC composites. Besides, some sintering additive may act as carbide binder (TiC, TaC, and SiC) or oxide binder (Al_2_O_3_, ZrO_2_, and Y_2_O_3_), which significantly improve the sinterability of tungsten carbide. On the other hand, with a deep understanding of brittle fracture, various toughening methods, for example transformation toughening, have been proposed to reduce the brittleness and enhance the strength and toughness of BTC.

Though BTC has been developed in various research groups during the past decades, there are still no comprehensive reviews of the processing–composition–microstructure–property relationships governing the material. In light of this, the present article aims to understand the fundamentals of sintering, toughening, and mechanical properties of BTC through a comprehensive review and a systematic examination of the processing, composition, microstructure, and mechanical properties, so as to provide adequate references for those who want to improve the performance of cemented carbides with better reliability and prepare the material in a way that is cost-effective, environmentally friendly, and benign to human health. The following review will be divided into three major sections: methods improving the sinterability of BTC including the design of mixed carbon content, chemical composition as well as advanced sintering techniques; strategies of toughening BTCs including particle dispersion toughening, transformation toughening, whisker toughening, carbon nanotube toughening, graphene toughening, and laminated structure toughening together with synergistic toughening; and mechanical properties of BTC including hardness versus fracture toughness coupled with wear resistance.

## Densification of Binderless Tungsten Carbide

Full densification is a prerequisite for BTC achieving its intrinsic properties. However, BTC, owing to the absence of any metallic binders, cannot be prepared by classical liquid-phase sintering but consolidated through solid-state sintering with higher temperature, i.e., above 1800 °C, as well as external pressure because of its low self-diffusion coefficients and strong covalent bonds [16–19]. Thus, it is essential to understand the fundamental of solid-state sintering. Solid-state sintering refers to a diffusional transport of matter through a particular path, which is driven by the reduction in surface free energy of the particle, activated by temperature, and dictated by chemical potential gradients associated with defect concentration in the solid. It can be divided into three stages [20, 21]. The initial stage features interface formation as well as neck growth between primary particles. It ends when the grain begins to growth. The intermediate stage features extensive grain boundary formations. Pores are still connected with each other in this stage. The final stage begins when pores become isolated. This stage features a fast grain growth with slight densification enhancement. Only four mechanisms are operative in solid-state sintering, and those are grain boundary diffusion, surface diffusion, vapor transport, and lattice diffusion [22]. With respect to solid-state sintering of BTC, grain boundary diffusion may play the critical role in the densification process. During the past decades, some efforts have been made to develop new sintering techniques as well as methodologies improving the sinterability of BTC, so as to obtain a fully dense BTC.

In 1957, Agte et al. [23] firstly attempted to fabricate pure WC employing conventional sintering techniques; however, the result was unsatisfactory. In 1990s, the availability of ultra-fine even nanopowders possessing increased surface energy, and some sintering actives shed light on the preparation of BTC through conventional sintering methods. It is revealed that the densification of nanocrystalline WC–Co powder took place mostly in solid state [24], suggesting that cobalt may not act significantly in the densification process of nanocrystalline WC–Co. Richter et al. [25–27] originally reported on the production of completely densified pure WC using vacuum or pressurized gas sintering owing to the utilization of ultra-fine WC starting powder. The author was appreciated by the EPMA Award of Merit for New Materials in the year of 2000 for developing an ultra-fined BTC with high hardness and wear resistance without any or very low amounts of grain growth inhibitor addition [28, 29]. Recently, some fast sintering techniques such as SPS featuring rapid heating and rapid sintering at relatively lower temperature further facilitated the fabrication of BTC. Fully densified BTC with fine grains is ultimately preferred and is critical to maximize properties. In the following, we critically analyze the major challenges involved in the densification of BTC, including various approaches required to overcome the sintering difficulty of BTC, and then effectively utilize the sintering factors to advantage the consolidation of BTC.

### Mixed Carbon Content

**Fig. 2:**
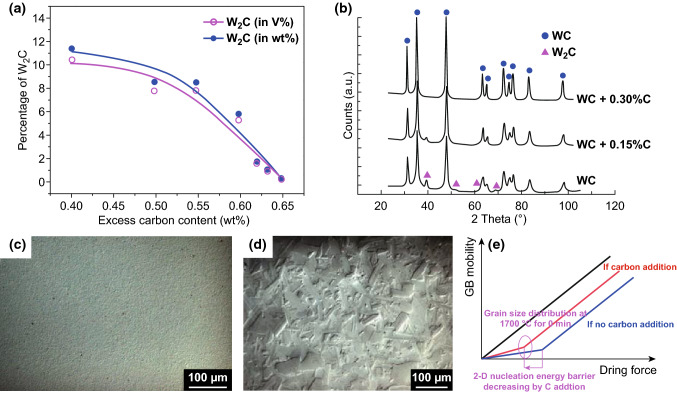
**a** Percentage of impurity phase W_2_C phase in sintered sample obtained by SPS at 1800 °C for 6 min under the pressure of 70 MPa as a function of C addition in powders [32]. **b** XRD patterns of WC micro and WC ball milled with carbon addition, **c** WC + 0.15%C and **d** WC + 0.3%C SPS 2200 °C without holding time [40]. **e** Schematic of abnormal grain growth due to addition of C [41]. Figure panels reproduced from Ref. [32] with permission from Taylor & Francis copyright 2015, Ref. [40] with permission from Elsevier copyright 2008, Ref. [41] with permission from Elsevier copyright 2003

Mixed carbon content has been of great concern to manufacturers of cemented carbide components since the 1950s [30, 31]. Adding free carbon into WC can improve the densification process and reduce or eliminate the formation of brittle phase W_2_C in samples obtained by SPS at 1800 °C for 6 min under the pressure of 70 MPa as shown in Fig. 2a. It is suggested that carbon addition is required to reduce oxide impurities as passivation layers located on the surface of WC grains and create the unifying phase compositions, ultimately obtaining single-phase WC materials. Gubernat et al. [33, 34] produced dense single-phase WC polycrystals by hot-pressing sintering with carbon addition. It was found that graphite nanolayers were formed on inter-granular boundaries in carbon-added WC sinters, which significantly improved the thermal conductivity of the polycrystals. The thermal conductivity of sintered WC samples without carbon addition was around 100 W/(m deg), whereas that of obtained WC specimens with carbon addition was close to 200 W/(m deg). However, excessive carbon addition can easily lead to the abnormal grain growth (AGG) of WC as well as the formation of graphite phase inducing cavities and pores in the sintered products [16, 35–38] preventing the full densification, implying that strictly controlling carbon content is essential in the design and fabrication of BTC.

As for conventional cemented carbides as WC–Co cemented carbides, there exists a carbon window, carbon content below which η-phase forms, whereas carbon content above which graphite phase precipitates. Unfortunately, no carbon range but only at a critical value carbon content can deleterious eta phase generation and graphite precipitation be avoided with respect to binderless cemented carbides [39]. Zhang et al. [39] synthesized pure WC employing SPS by tailoring carbon addition to the starting powders. In case of WC with an initial powder size of 0.2 μm, the sintered product was a single WC phase only with a 0.4 wt% carbon mixed. WC_1-x_ phase formed regarding mixed carbon content below 0.4 wt%, while free carbon appeared concerning mixed carbon content above 0.4 wt%. The main phase changed into WC_1-X_ and W_2_C for samples without carbon addition. The authors stated that higher mixed carbon content was required for fine-grained BTC with a correct phase in comparison with conventional cemented carbides as WC–Co. For one thing, more oxygen was absorbed by WC powders with fine grade (with larger-surface-to-volume ratio). Besides, higher sintering temperature (about 1800 °C) contributed to a complete oxide reduction process. Girardini et al. [40] prepared WC with commercially available micro-WC powders employing SPS with a sintering temperature of 2200 °C and no holding time. With 0.15 wt% carbon addition, the sintered samples appeared no AGG (Fig. 2c), but the W_2_C phase was detected as shown in Fig. 2b, implying that the added carbon was insufficiently used for complete oxide reduction. With 0.3 wt% carbon addition, the W_2_C phase was avoided (Fig. 2b); however, no improved mechanical properties were obtained as a result of the occurrence of AGG as illustrated in Fig. 2d. Single-phase WC products can be obtained through adding carbon, but AGG occurred once the carbon content exceeded the stoichiometric amount required for the reduction of oxides. In other words, carbon addition reduces or eliminates the brittle phase W_2_C, and this leads to an enhancement in mechanical properties as fracture toughness; however, at the same time carbon contributes to the AGG and this second effect depresses the mechanical properties as hardness and flexural strength of the material.

As for this, the densification and grain growth behavior of binderless WC are sensitively dependent on the C content within WC. It is rather challenging to strictly control the carbon content during the fabricating process, and the correlation between carbon content and AGG must be carefully taken into account for the design and fabrication of BTC.

Cha et al. [41] reported that W_2_C formation was attributed to the carbon loss through interaction of carbon with surface oxides. Carbon addition was necessary for reduction of oxide impurities located mainly as passivation layers on the surface of WC grains. It is proposed that the densification and grain growth behavior of BTC are sensitively dependent on the C content within tungsten carbide. WC with an initial powder size of 0.57 μm could be sintered to densification by addition of 0.3 wt% free carbon. The authors also demonstrated the mechanism for carbon addition-induced AGG as shown in Fig. 2e. From their perspective, the carbon addition can not only increase the driving force of grain growth, but also decrease the two-dimensional nucleation energy barrier in WC; as a consequence, the two-dimensional nucleation insufficiently restricts the grain growth due to the sufficiently large driving force for grain growth. Zhao et al. [42] prepared highly dense BTC with a grain size of around 0.4 μm by the SPS method. The authors found that the carbon addition-induced AGG occurred only when the sintering temperature was above 1650 °C. To avoid AGG, specimens were consolidated at a lower temperature 1500 °C with a holding time 5 min, the density was around 99%, and no AGG occurred. On the other hand, based on the well-known equations as Eqs. 1–6, it is suggested that the excess carbon in WC can be moderated by introducing proper contents of WO_3_ and W [39, 43].

In the presence of free carbon:

1 $${\text{W}} + {\text{C}} \to {\text{WC}}$$

2 $${\text{WO}}_{3} + 4{\text{C}} \to {\text{WC}} + 3{\text{CO}}\,\left( {\text{g}} \right)$$

3 $$2{\text{WO}}_{3} + 5{\text{C}} \to 2{\text{WC}} + 3{\text{CO}}_{2} \,\left( {\text{g}} \right)$$

In the absence of free carbon:

4 $${\text{W}} + {\text{WC}} \to {\text{W}}_{2} {\text{C}}$$

5 $${\text{WO}}_{3} + 7{\text{WC}} \to 4{\text{W}}_{2} {\text{C}} + 3{\text{CO}}\,\left( {\text{g}} \right)$$

6 $$4{\text{WO}}_{3} + 16{\text{WC}} \to 10{\text{W}}_{2} {\text{C}} + 6{\text{CO}}_{2} \left( {\text{g}} \right)$$

In summary, carbon control is very essential for BTC achieving maximum densification and performance levels. Strict design of additives including carbon, WO_3_, and W together with careful control of thermal processing process is of great importance to the carbon content in the final product.

### Nanocrystalline Tungsten Carbide and Its Composites

**Fig. 3:**
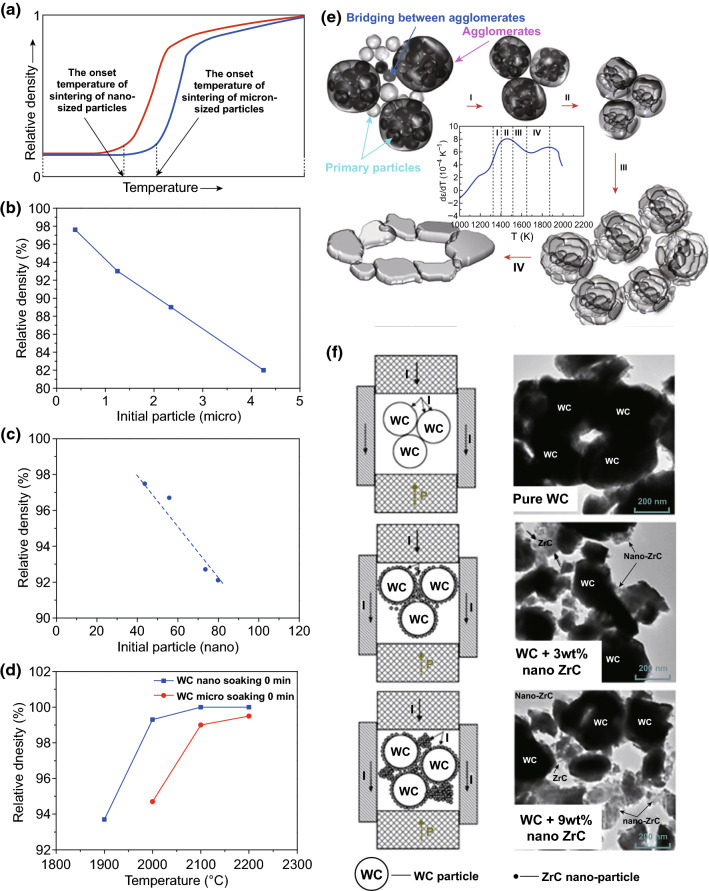
**a** Schematic diagram illustrating different onset temperatures of sintering of nano- and micro-sized particles [44]. Relative density of pure WC as a function of initial particle size in BTC **b** microsize [56] and **c** nanosize [57]. **d** Relative density of micro and nano WC versus sintering temperature, without holding time [40]. **e** Schematic of the sintering mechanism of nano WC on the basis of the experimental shrinkage strain rate in a non-isothermal experiment [48]. **f** Schematic diagrams and corresponding TEM micrographs revealing the contact state of WC and ZrC nano-particles during sintering [63]. Figure panels reproduced from Ref. [44] with permission from Taylor & Francis copyright 2008, Ref. [56] with permission from Elsevier copyright 2004, Ref. [57] with permission from Elsevier copyright 2017, Ref. [40] with permission from Elsevier copyright 2008, Ref. [48] with permission from Elsevier copyright 2011, Ref. [63] with permission from Elsevier copyright 2015

Based on various sintering models, from the two sphere ones for the initial sintering stage to the microstructural model for the intermediate sintering stage, sintering temperature strongly depends on the grain size, that is, finer initial powder grain size is helpful in lowering the sintering temperature or starting temperature as well as shortening the sintering process, especially for nanopowders as illustrated in Fig. 3a [44–51]. It is demonstrated that nanosized WC–Co powders start to densify at 600 °C, while submicron-sized powders begin at 1100 °C and regular micro-sized powders at 1320 °C (WC–Co liquidus point) [52–54], conforming the profound effect of grain size on the onset of sintering of WC–Co cemented carbides. Muginstein [55] reported that submicron-sized WC powders begin to densify at 1200 °C, while nanopowders at 500 °C, suggesting that grain size also acted significantly during the sintering of BTC. Furthermore, according to the interfacial energy considerations, there exists a critical pore coordination number, only below which pores will shrink and disappear; otherwise, pores will be stable and not shrink during the sintering process. The grain growth of nanocrystalline particles is helpful in lowering the coordination number of pores and then contributing to the densification process. Also, in general, the average pore size is strongly associated with the average particle size, so nanocrystalline particles may lead to nanosized pores with higher mobility, in turn accelerating the densification process.

Kim [56] and Chvildeev [57, 58] investigated in detail the effect of initial WC particle size on the densification of pure WC by determining the relative densities of sintered BTC with varied initial particle sizes after the same sintering condition. As shown in Fig. 3b, c, the relative density of BTC increased with decreased initial particle size. Therefore, reducing the particles size even down to nanorange is feasible to improve the consolidation process. Girardini et al. [40] compared the densities of micro- and nano-WC with increasing sintering temperature as shown in Fig. 3d. Obviously, there exists a densification delay for micro-WC powder in comparison with nano-WC powder, indicating that nano-WC powder possesses better sintering activity [24, 59–61]. The driving force for densification of WC in general results from a reduction in total surface energy. The internal interface area increased with a decrease in WC grain size, and an enlarged interface area is essential to an enhanced sintering driving force. Based on conventional sintering theories, the sintering driving force can be calculated by Laplace equation (Eq. 7):

7 $$\sigma = \gamma \left( {\frac{1}{{R_{1} }} + \frac{1}{{R_{2} }}} \right)$$

where *γ* is the surface energy of the material and *R*_1_ and *R*_2_ are the principal radii of the curvature. Obviously, nanocrystalline WC powders possess much higher sintering driving forces in comparison with micro-sized ones, due to their superior surface energy. It is concluded that nanocrystalline WC powders possess better sinterability in comparison with micro-sized ones, due to their high surface energy increasing the sintering driving forces.

By investigating sintering kinetics of nanocrystalline WC powders (100–500 nm), Muginstein [62] defined three sintering stages regarding the nanocrystalline WC powder: (1) initial stage, rearrangement of particles at low temperature (850 °C) with no grain or particle growth; (2) intermediate stage, neck formation between powder particles at 1000–1250 °C and initial grain growth at 1200 °C; and (3) final stage, pore elimination accompanied by massive grain growth at 1300–1450 °C.

The sintering kinetics of nano-grained WC was studied in detail by Nanda Kumar et al. [48] using commercially available nano-WC powder without any pretreatment with particle sizes measuring 70 nm (by BET analysis) employing non-isothermal and isothermal sintering. The results illustrated that the agglomeration of nanoparticles was easily to be brought about by surface diffusion resulting in neck growth and grain rotation even at low temperature. The sinterability differences of inter- and intra-agglomerate pore phases introduced substages into the intermediate stage during the sintering process as shown in Fig. 3e. Four substages were defined in the intermediate sintering stage: (1) stage I (~ 80% end density), this stage was located at the end of initial sintering stage/starting of the intermediate sintering stage and covered the temperature from 1027 to 1127 °C. This stage featured a rapid increase in the densification rate, resulting from inter-agglomerate together with intra-agglomerate densification. Most bridges between agglomerates disappeared and then clusters impinged on each other. At the end of this stage, the microstructure evolved into most agglomerate and continuous pore; 2) stage II (~ 83 to 85% end density), this stage was identified for its constant density. The shrinkage, if any, occurred purely as a function of intra-agglomerate densification. At the end of this stage, the microstructure was characterized by well-demarcated agglomerate boundaries; 3) stage III (~ 85% end density), in this stage, the net shrinkage stain rate decreased owing to inter-agglomerate pore stability as well as a small amount of grain growth within agglomerates; (4) stage IV (~ 94% end density), in this stage, because of temperature driven coupled with the reduction in pore coordination number, the system tried to evolve by massive grain growth within the agglomerates, leading to the breakup of the agglomerate and sintering start of stable inter-agglomerate pores. The pores started to shrink rapidly and surpassed the grain growth rate with the transform of agglomerates into large grains. Thus, in nano-WC system, the grain growth acted to enhance the densification process by changing the pore stability from thermodynamically stable to unstable through decreasing the pore coordination number. Actually, considering the thermodynamics of the sintering process, complete densification cannot be achieved in nano-WC powders without grain growth as one abets the other [48].

Besides reducing the particle size of WC matrix, it is also effective to facilitate the sintering of WC by dispersing some nanosized second-phase additive within micro-sized WC matrix grains or at the grain boundaries of WC matrix. Ren et al. [63] reported that the densification of BTC was significantly improved through adding appropriate amount of ZrC nano-powder. The densification mechanism was that submicron-sized WC particles were surrounded by nanosized ZrC particles through the mixing process as shown in Fig. 3f. WC particles tended to gather together to form a large particle regarding pure WC sample. However, with respect to 3 wt% nano-ZrC-added specimen, submicron-sized WC particles were adhered by nano-ZrC particles (Fig. 3f). The adherent nano-powder filled the inter-space resulting from particle rearrangement and plastic deformation of WC grains and then improved the densification process. On the other hand, nanopowders tended to agglomerate as a result of their high specific surface areas. Considering the WC–9 wt% ZrC samples, the agglomeration of nano-ZrC was serious, consequently leading to the deterioration of densification.

Biswas [64] prepared fully dense WC–6 wt% ZrO_2_ composites with the assistance of ZrO_2_ powders of 27 nm as second-phase employing SPS (sintering at 1300 °C for holding 5 min). As demonstrated in Table 2, employing the same consolidation technique, sintering temperature required for WC achieving full densification was significantly reduced as a function of nanocrystalline ZrO_2_ addition. Furthermore, it should be noted that WC can be consolidated utilizing nanosized ZrO_2_ at a lower temperature 1300 °C in comparison with WC–Co at 1400 °C [65], indicating that nano-ZrO_2_ is an ideal alternative to Co binder as sinter additive. The densification improving mechanism was ZrO_2_ enhancing sintering kinetics as a result of size effect together with high “constriction resistance.” Nanosized ZrO_2_ increased the volume fraction of the interface area due to its higher specific surface area and then resulted in faster mass transport considering grain boundaries providing faster diffusion paths. Nanosized ZrO_2_ particles enhanced “constriction resistance,” because of their nanosize, resulting in higher resistivity coupled with smaller particle-to-particle contact area, based on Eq. 8 [66]:

8 $$R_{\text{c}} (n,a,l) = \frac{{\rho f_{\text{s}} }}{2\pi na}\arctan \left( {\frac{{\sqrt {l^{2} - a^{2} } }}{a}} \right)$$

where *R*_c_ is the constriction resistance, *ρ* is the resistivity of the powder, *a* is the linear dimension of the inter-particle contact, *f*_s_ is the shape factor, *l* is the nearest distance between the contacts, and *n* is approximately the number of squares with sides 2*l*. Thus, voltage drop took place over a small distance around the nano-ZrO_2_ contact area. High-resistance localized area with a rather high Joule heating was formed, resulting in local temperature increase and then a faster mass transport.

**Table 2:** Densification data of WC–6 wt% ZrO_2_ composites and WC–6 wt% Co cemented carbides sintered via spark plasma sintering (SPS)

Material composition	Starting powder size (µm)	Sintering techniques and temperature (°C)	Holding time (min)	Pressure (MPa)	Relative density (%)
WC–6 wt% ZrO_2_ [64]	0.2	SPS, 1300	5	30	99.9
WC–6 wt% Co [65]	0.2	SPS, 1300	5	30	91.4
WC–6 wt% Co [65]	0.2	SPS, 1400	5	30	100

### Ceramic Sintering Additive

Sintering additives, which are present in solid state only or may form liquid phases, are intentionally added to control the microstructural and dimensional development during sintering. Numerous researches have demonstrated that minor additions of second phase, which increase the grain boundary diffusion and surface diffusion in this system, accelerate the densification process [67]. Therefore, besides the mixed carbon content and carbide grain size, the design of chemical composition is also a logical strategy to enhance the sinterability of tungsten carbide in conventional methods. The low melting point of the metal and possibility of carbonation and oxidation make the selection of the transition-metal carbides (TiC [68, 69], TaC [70], and SiC [71, 72]) and metal oxides (Al_2_O_3_ [73], ZrO_2_ [74], Y_2_O_3_ [75, 76], and La_2_O_3_ [77]) binders more favorable for tailoring the densification process of BTC.

#### Titanium Carbide (TiC)

Titanium carbide (TiC), with a cubic structure (Ti atoms on one fcc sublattice and C atoms on the other), has been recognized as one of the most significant metal carbides for BTC manufacturing, due to its prominent intrinsic properties, such as high melting point (3067 °C), high thermal conductivity (30 × 10^6^ S cm^−1^), high hardness (TiC has 31 GPa Vickers hardness and WC has 25 GPa), high elastic modulus (410–450 GPa), high thermodynamic stability together with low friction coefficient and density (4.93 g cm^−3^) [78]. In 1982, Kanemitsu et al. [15] firstly developed a WC–TiC–TaC binderless cemented carbide. Since then, several researches have been performed to improve the sinterability of tungsten carbide through adding TiC as a carbide binder.

**Fig. 4:**
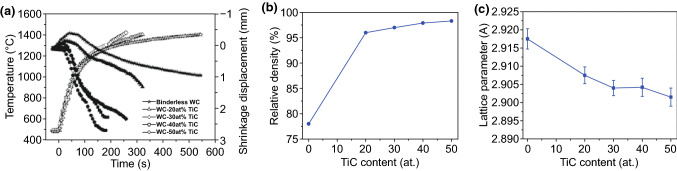
**a** Variations of temperature and shrinkage displacement with heating time during high-frequency induction heated sintering of WC–*x* at.% TiC hard materials (under 60 MPa pressure, 2800A). **b** Relative density as a function of TiC content. **c** Plot of WC phase lattice parameter as a function of TiC content produced by PCAS [79]. Figure panels reproduced from Ref. [79] with permission from Hanyang University copyright 2007

Kim et al. [79] demonstrated the effect of TiC content on the sintering of WC–TiC composites as illustrated in Fig. 4. Initially, all specimens showed volume increases because of the thermal expansion. The onset of shrinkage of pure WC started at about 1200 °C, while that of TiC-added WC composites took place at around 950–1100 °C, suggesting that the content of TiC had a significant influence on the onset of sintering of WC-based composites. Sintering temperature was decreased significantly with the increasing TiC content. As depicted in Fig. 4b, under the same sintering condition, the density of pure WC sample was about 78%, while that of WC–50 at.% specimen was 98.5%. Obviously, TiC was beneficial to enhance the relative density of WC–TiC composites. The densification mechanism was that TiC acted as a carbide binder resulting from the formation of WC–TiC solid solution phase [80–83]. As shown in Fig. 4c, the lattice parameter of WC decreased with the increasing contents of TiC addition, confirming the formation of a series of solid solution.

It was thought that the formation of solid solutions between additive and WC could facilitate the densification of WC-based materials. The similar densification mechanism was found in WC–TiC–TaC [80], WC–VC [84], and WC–Mo_2_C [85] systems. Imasato et al. [80] reported that the addition of TaC and TiC facilitated the sintering process of WC because of the formation of (W, Ta, Ti) C solution phase. Huang et al. [84] suggested that VC addition facilitated densification as a function of (V, W) C solid solution phase formation, so that full densification for WC–VC composites could be achieved at lower temperature or within shorter sintering time in comparison with pure WC.

#### Silicon Carbide (SiC)

Silicon carbide (SiC) possesses a high hardness above 20 GPa together with a relatively high Young’s modulus 440 GPa. Furthermore, SiC is less expensive than WC. Additionally, SiC is proved to be a toughening phase for many ceramics including WC. So, SiC can be an excellent candidate for improving the sinterability of BTC. It is proposed that the sintering temperature of WC was significantly decreased as a function of SiC powder and/or SiC whiskers addition [86–88].

**Fig. 5:**
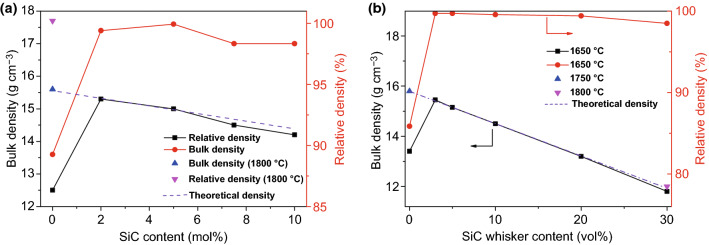
**a** Bulk density and relative density of WC–SiC composites [87]. **b** Bulk density and relative density of WC–SiC_w_ composites [88]. Figure panels reproduced from Ref. [87] with permission from Japan Institute of Metals (JIM) copyright 2011, Ref. [88] with permission from Japan Institute of Metals (JIM) copyright 2008

Dense WC–SiC composites were fabricated by Nino et al. [87] employing hot-pressing sintering methods. As demonstrated in Fig. 5a, no dense sintered body was obtained at 1600 °C for pure WC though hot-pressing applied, indicating that 1800 °C was essential to realize densification. However, with respect to WC–SiC composites, high relative density above 98% was achieved as a result of 2–10 mol % SiC addition.

As shown in Fig. 5b, WC–SiC_w_ composites containing 3–5 vol% SiC_w_ were fully dense at 1650 °C, while pure WC could not be sintered densely at 1650 °C, suggesting that SiC_w_ was efficacious in lowering the sintering temperature of WC [88]. However, the relative density gradually decreased with the SiC_w_ addition regarding 10 vol% SiC_w_. 1750 °C was necessary for 30 vol% SiC_w_ composites to realize a fully dense level.

However, while initially appealing, caution should be exercised as the mechanism of the WC sinterability improvement by SiC addition, to data, is still unclear and WC–SiC composites, possessing only a small market share, were employed only to a limited extent for specialized applications. Moreover, it is noted that SiC addition tended to result in the abnormal growth of WC grains; thus, some grain growth inhibitors such as ZrC [71], NbC [72], Mo_2_C [89], VC [56], and Cr_3_C_2_ [86, 90] are required to obtain the excellent microstructure as well as mechanical properties.

#### Alumina (Al_2_O_3_)

Alumina (Al_2_O_3_), as a refractory oxide, has been widely used in industry applications owing to its excellent properties, such as low density, good thermal and chemical stability, high hardness, and high temperature [91]. Besides, the price of Al_2_O_3_ is lower than that of Co and many other metal oxides as ZrO_2_ and MgO. Moreover, Al_2_O_3_ has a lower melting point in comparison with that of ZrO_2_ and MgO, which is more helpful in lowering the temperature of reinforced materials by generating liquid phase. Therefore, Al_2_O_3_ can be an economical and suitable candidate to replace Co in WC matrix [92–99].

**Fig. 6:**
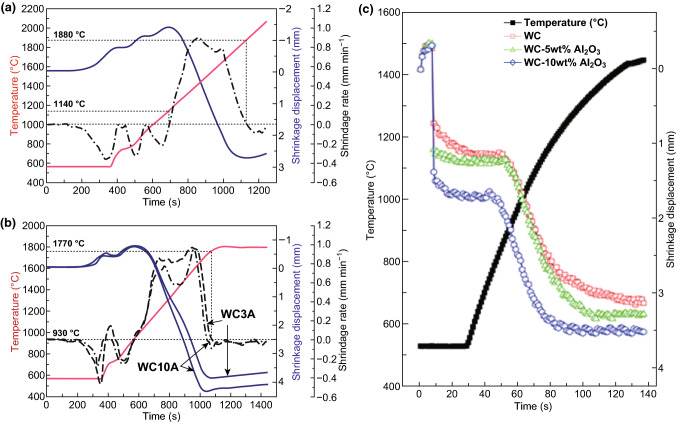
Densification behavior of **a** WC, SPSed at 2060 °C for 0 min, and **b** WC3A (WC–3 wt% Al_2_O_3_) and WC10A (WC–10 wt% Al_2_O_3_), SPSed at 1800 °C for 5 min [99]. **c** Variations of temperature and shrinkage with heating time during the pulsed current activated sintering of WC–Al_2_O_3_ powder [100]. Figure panels reproduced from Ref. [99] with permission from Elsevier copyright 2012, Ref. [100] with permission from Elsevier copyright 2016

Zheng et al. [99] investigated the effect of Al_2_O_3_ addition on the sintering behavior of WC–Al_2_O_3_ composite as shown in Fig. 6a, b. For pure WC, the densification process took place at around 1140 °C and ended at about 1880 °C. As far as WC–3 wt% Al_2_O_3_ was concerned, the densification starting point was ~ 930 °C and the ending temperature was ~ 1770 °C. Obviously, Al_2_O_3_ addition significantly facilitated the sintering process by accomplishing the densification process at a lower temperature range owing to Al_2_O_3_’s lower melting point of around 2050 °C than WC (about 2785 °C), which is well in line with the findings of Oh et al. [100] as depicted in Fig. 6c.

Qu et al. [101] prepared highly dense WC–(10, 20, 30, 40, 50) vol% Al_2_O_3_ composites by a hot-pressing sintering method, suggesting that the relative density increased with the increasing content of Al_2_O_3_. Furthermore, the author demonstrated that Al_2_O_3_ addition was effective to suppress the WC grain growth. A work conducted by Seung-Jin Oh et al. [102] reported the same conclusion through HFIHS methods as demonstrated in Table 3.

**Table 3:** Average grain size and relative density of pure WC and WC–Al_2_O_3_ composite after the same HFIHS sintering conditions with same staring WC powders [102]

Composition	Relative density (%)	Grain size (nm)
WC	97	265
WC–5 vol% Al_2_O_3_	98	185
WC–10 vol% Al_2_O_3_	99	138
WC–15 vol% Al_2_O_3_	99.8	101

#### Zirconia (ZrO_2_)

Zirconia (ZrO_2_) has received attention as a substitute for Co because of its excellent properties: (1) high chemical stability up to quite high temperature about 1400 °C; (2) not softening at elevated temperatures; (3) high electrochemical corrosion resistance; (4) increasing toughness by transformation toughening [103–107]. Besides, ZrO_2_ was found to facilitate the sintering of BTC by many researches [108–110].

**Fig. 7:**
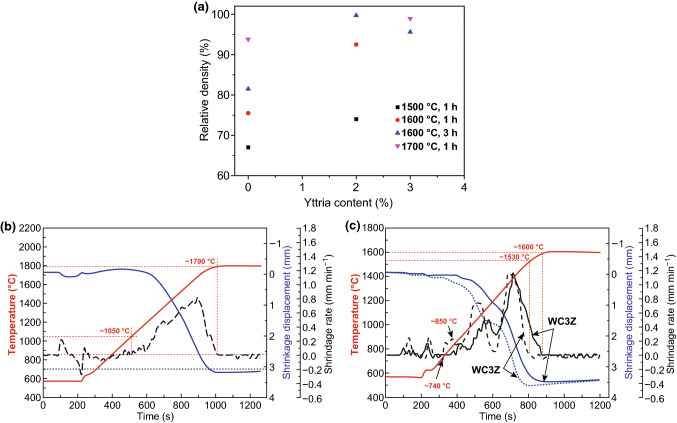
**a** Relative density obtained at different sintering temperatures with WC composites with 6 wt% ZrO_2_ (stabilized with varying amount of Y_2_O_3_) sinter-additive [111]. Densification curves for **b** pure WC sintered at 1800 °C for holding 5 min, **c** WC3Z and WC10Z specimens sintered at 1600 °C for holding 5 min [104]. Figure panels reproduced from Ref. [111] with permission from Elsevier copyright 2005, Ref. [104] with permission from Elsevier copyright 2013

Basu et al. [111] proposed that it was inappropriate to use ZrO_2_ itself as the sintering additive, but Y_2_O_3_-stabilized tetragonal ZrO_2_ was effective to improve the sinterability of BTC. Figure 7a illustrates the effect of Y_2_O_3_ content in ZrO_2_ on the densification of WC employing pressureless sintering. Though sintered at 1700 °C for 1 h, the composites can only achieve a relative density of 93.9% with Y_2_O_3_-free ZrO_2_ addition, conforming that Y_2_O_3_-free ZrO_2_ was ineffective to enhance the densification process of WC.

Figure 7b, c demonstrates the densification curves for the pure WC sintered at 1800 °C for holding 5 min and the WC3Z (WC–3 wt% ZrO_2_) and WC10Z (WC–10 wt% ZrO_2_) samples sintered at 1600 °C for holding 5 min. Caution should be noted that the ZrO_2_ used in this research was 3 mol % Y_2_O_3_-stabilized tetragonal ZrO_2_. Ignoring several shrinkage rate fluctuations below ~ 650 °C, it is considered that the densification process took place when the shrinkage rate turned to be positive and ended when the shrinkage rate was down to zero again. As shown in Fig. 7b, the densification of pure WC started at around 1050 °C and ended at around 1790 °C. Concerning WC sample, the densification began at a lower temperature ~ 850 °C and ended at a lower temperature ~ 1600 °C in comparison with pure WC. Further increasing ZrO_2_ content, the densification process can be accomplished at even lower temperature range. With respect to WC10Z specimen, the densification beginning temperature yielded at ~ 740 °C, and the ending point of densification was ~ 1530 °C. Obviously, ZrO_2_ addition significantly improved the densification process of WC. Additionally, the cures exhibited a bimodal shape regarding WC–ZrO_2_ specimens, an extra shrinkage rate maximum appearing at about 1050–1100 °C, which was considered as a result of transformation of retained m-ZrO_2_ in original ZrO_2_ powders involving a volume decrease (3–5%) and assisting particle rearrangement during the densification process. Since the densification process is mainly diffusion controlled, it is denoted that ZrO_2_ addition elevated the diffusivity of WC–ZrO_2_ system. Densification starting temperature was decreased due to ZrO_2_ addition, so the densification improving mechanism can be concluded that ZrO_2_ addition lowers the activation energy for atoms diffusion, resulting in a higher diffusivity and then an enhanced densification [104].

### Advanced Sintering Techniques

**Fig. 8:**
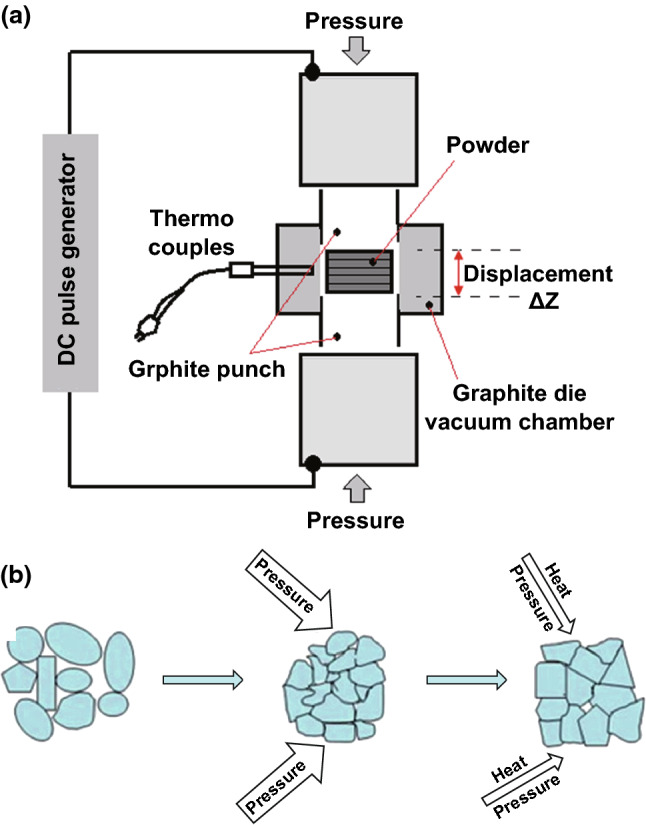
**a** Schematic representation of SPS equipment [112]. **b** The formation mechanism of WC sintered under high pressure [18]. Figure panels reproduced from Ref. [112] with permission from Taylor & Francis copyright 2007, Ref. [18] with permission from Elsevier copyright 2016

The properties of BTC fabricated by powder metallurgy can be significantly enhanced through improving the density coupled with decreasing the grain size of the sintered bodies. However, since WC possesses a high melting point (2785 °C) and no metallic binder addition, it is rather difficult to consolidate WC to a high density with a fine grain employing the conventional sintering process. Generally speaking, excessively high sintering temperature and/or long holding time is easy to incur porosities in the sintered material and results in coarsening of the microstructure. Thus, it is crucial to carefully control the sintering parameters, particularly sintering temperature and time, for preparing materials with excellent properties. Compared with conventional sintering techniques, some advanced sintering techniques such as fast sintering techniques are known for their higher heating and cooling rates, as well as shorter densification time at lower sintering temperature, as a function of external field together with high-pressure application such as SPS, RSPS, PCAS, and HFIHS. SPS, for example, applying pulsed electrical fields coupled with resistance heating and pressure as shown in Fig. 8a, can consolidate materials rapidly, uniformly, and thoroughly because of the large pulsed direct current during heat treatment of powders in the graphite die. The external field is prone to enhancing the mass transport during the sintering, and the effect can be determined through the electromigration theory (Eq. 9) [113]:9where *J*_*i*_ is the flux of the diffusing ith species, *D*_*i*_ is the diffusivity of ith species, *C*_*i*_ is the concentration of ith species, *F* is Faraday’s constant, *z*^*∗*^ is the effective charge on the diffusing species, *E* is the field, *R* is the universal gas, and *T* is absolute temperature. Furthermore, high pressure is helpful in accelerating the particle rearrangement, sliding, or even distortion as well as breaking (Fig. 8b). These merits of fast sintering techniques highly open up the possibilities for successful production of highly dense BTC with fine grains.

Table 4 summarizes the densification as well as grain sizes of materials obtained through various sintering techniques. Obviously, even without any sintering additive or grain growth inhibitor addition, fine-grained WC with nearly full densification was prepared. SPS, the most employed sintering technique during the fabrication of BTC, attracted most attention among materials researchers as well as production engineers. SPS was proposed according to the idea employing the plasma on electric discharge machine for sintering ceramics and metal in the early 1960s by Inoue et al. [114] expecting that the plasma-assisted sintering could contribute to the realization of advanced materials [115]. Thereafter, the concept obtained further development during the mid-1980s to the early 1990s. In 1998, it was first experimentally verified that SPS enhanced the densification of materials [116]. SPS is also named as pulsed electric current sintering (PECS) or electric pulse-assisted consolidation (EPAC) [117, 118]. Conventional rapid heating can easily result in temperature gradients and then differential densification (non-uniform microstructures), low density, or sample cracking. SPS densifies the materials at a homogeneous temperature or heat distribution by applying pulsed direct current through the sample and graphite die. Furthermore, it is found by Huang et al. [119] that the grain growth of WC hardly occurred during SPS process, which is greatly different from WC–Co cemented carbides showing obvious WC grain coarsening during SPS process [120].

**Table 4:** Results of various sintering techniques regarding densification as well as grain size

Sintering process	Temperature and holding time	Heating rate (°C min^−1^)	Pressure (MPa)	Starting powder and grain size	Relative density (%)	Sintered grain size (nm)
HP^a^ [33]	2150 °C/1 min	N/A	25	WC, 0.5–0.7 μm	97.5	
HP [121]	2000 °C/30 min	3-10	28	WC–0.33 wt% VC–0.54 wt% Cr_3_C_2_, 0.53 μm	97.9	730
HIP^b^ [121]	1750 °C/120 min	N/A	150	WC–0.33 wt% VC–0.54 wt% Cr_3_C_2_, 0.53 μm	99.4	750
HP [90]	1600 °C/10 min	50	50	WC–20 mol% SiC, 0.73 μm	98	530
HP [90]	1600 °C/10 min	50	50	WC–20 mol% SiC–0.9 mol% Cr_3_C_2_, 0.73 μm	98	340
HP [101]	1540 °C/90 min	10	39.6	WC–40 vol% Al_2_O_3_	97.98	2750
HPHT^c^ [18]	1500 °C/20 min	100	5 GPa	WC, 150–250 nm	99.2	150-250
GPS^d^ [122]	1860 °C/60 min	3-6	0	WC, 1.03 μm	95.1	
SPS^e^ [119]	1500 °C/4 min	180	60	WC, 200 nm	99.6	200
SPS [123]	1460 °C/3 min	150	30	WC–0.1 wt% C, 200 nm	99.5	350
RSPS^f^ [124]	1350 °C/1.5 min	100	60	W_2_N-C, 40 nm		270
SPS [125]	1800 °C/1 min	150	80	WC, 55 nm	100	160
SPS [126]	1750 °C/10 min	50	50	WC, 1.8 μm	99.6	
SPS [42]	1750 °C/0 min	1500	126	WC, 40–70 nm	100	305
SPS [127]	1400 °C/2 min	180	50	WC–0.3 wt% VC–0.5 wt% Cr_3_C_2_, 200 nm	97.5	240
SPS [63]	1600 °C/5 min	105	50	WC–3 wt% ZrC, 700 nm	100	
SPS [64]	1300 °C/5 min	600	30	WC–6 wt% ZrO_2_, 200 nm	99.9	
PECS^g^ [83]	1900 °C/1.5 min	200	60	WC, 200 nm	100	280
PECS [109]	1700 °C/1.5 min	200	60	WC–5 vol%ZrO_2_, 200 nm	100	
PCAS^h^ [100]	1450 °C/0 min	900	80	WC–10 vol% Al_2_O_3_, < 0.5 μm	98	112
SPS [128]	1450 °C/0 min	100	30	WC–2.8 wt% Al_2_O_3_–6.8 wt% ZrO_2_, 0.8 μm	99	
HFIHS^i^ [129]	1500 °C/100 s	1400	60	WC, 400 nm	98.5	380
HFIHS [130]	1250 °C/0 min	N/A	80	WC, 40 nm	99	87

^a^Hot pressing; ^b^Hot isostatic pressing; ^c^HIgh pressure and high temperature; ^d^Gas protection sintering; ^e^Spark plasma sintering; ^f^Reactive spark plasma sintering; ^g^Pulsed electric current sintering; ^h^Pulsed current activated sintering; ^i^High frequency induction-heated sintering

## Toughening of Binderless Tungsten Carbide

Brittle fracture limits the use of BTC in many applications. The fracture toughness of BTC strongly depends on the interaction of the crack tip stress field with the microstructure. Therefore, microstructural design to reduce stresses near crack tips has been a key issue during the development of highly tough BTC.

In order to reduce the brittleness and to enhance the strength and the toughness of BTC, varieties of traditional toughening methods, including particle dispersion toughening, transformation toughening, whisker toughening, and synergistic toughening, have been proposed in the past decades. Recently, with our deep understanding of brittle fracture, some new-concept toughening methods, such as laminated structure toughening, carbon nanotube toughening as well as graphene toughening, are proved to be also operative in BTC, offering great promise of a revolutionary advance in the production of high-toughness BTC. The purpose of this section is to demonstrate how the knowledge gained is currently being applied to the development of highly tough BTC or BTC composites.

### Particle Dispersion Toughening

Particle dispersion toughening, as the simplest toughening method, is the foundation of the other toughening methods of ceramics [131]. It can be subdivided into non-transformation secondary-phase particle dispersion toughening, ductile particle dispersion toughening as well as nanoparticle dispersion toughening. The major toughening mechanisms existing particle-toughened ceramics were ascribed to (i) crack deflection by the particulates ahead of a propagating crack (crack deflection model); (ii) crack bridging by particulates (particulate bridging model); (iii) interaction between the crack front and particulates (crack front bowing model); and (iv) thermal residual stress field resulting from mismatch in coefficients of thermal expansion of ceramic matrix and particulates as well as (v) the grain size [132, 133].

**Fig. 9:**
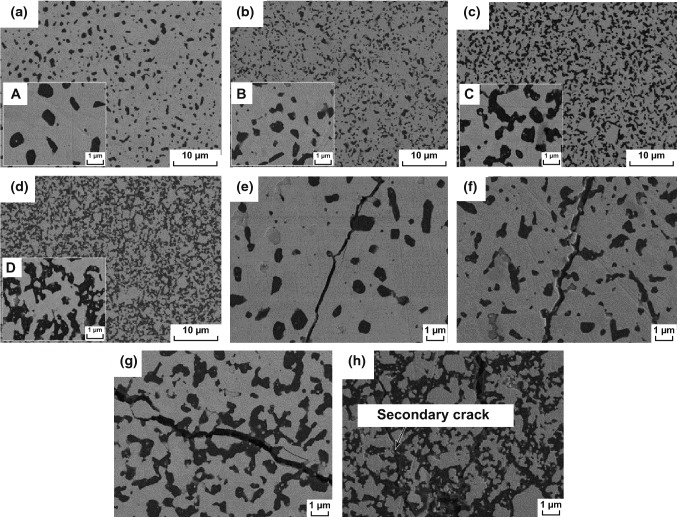
FE-SEM images of the polished samples showing the different dispersion states and morphologies of the toughening particulates: (**a, A**) WC–10 vol% Al_2_O_3_, (**b, B**) WC–20 vol% Al_2_O_3_, **(c, C**) WC–30 vol% Al_2_O_3_, and **(d, D**) WC–40 vol% Al_2_O_3_, composites sintered at 1540 °C for 90 min. FE-SEM images of Vickers indentation crack extension path of **e** WC–10 vol% Al_2_O_3_, **f** WC–20 vol% Al_2_O_3_, **g** WC–30 vol% Al_2_O_3_, and **h** WC–40 vol% Al_2_O_3_ composites sintered at 1540 °C for 90 min [101]. Figure panels reproduced from Ref. [101] with permission from Elsevier copyright 2012

With respect to BTC, the most used dispersed phases are Al_2_O_3_ [95, 99], MgO [134–137], TiC [79], SiC [71, 87, 90], Mo_2_C [85], and ZrC [63]. Qu et al. [101] successfully prepared WC–Al_2_O_3_ composite by employing conventional hot-pressing sintering. Al_2_O_3_ grains were homogenously distributed in WC matrix as demonstrated in Fig. 9. Excellent fracture toughness of 10.43 MPa m^1/2^ was obtained for WC–40 vol% Al_2_O_3_ composite. It is noted from Table 5 that significant toughening effect was achieved due to the dispersed Al_2_O_3_ particle. Figure 9e–h illustrates the Vickers indentation crack extension path of WC–Al_2_O_3_ composites. With the increasing Al_2_O_3_ content, the crack propagation path became more tortuous, and more crack bridgings and crack deflections were observed. Additionally, WC–40 vol% Al_2_O_3_ composites exhibited secondary crack pattern as depicted in Fig. 9h. The generation and existence of secondary crack consumed the energy of main crack and increased the length of crack propagation path, resulting in improved fracture toughness.

**Table 5:** Composition proportion, relative density and fracture toughness of WC–Al_2_O_3_ composites [101]

WC (vol%)	Al_2_O_3_ (vol%)	Sintering process	Relative density (%)	Fracture toughness K_IC_ (MPa m^1/2^)
90	10	1540 °C 90 min 39.6 MPa	95.73	7.86
80	20		96.52	8.10
70	30		96.53	9.43
60	40		97.98	10.43

**Fig. 10:**
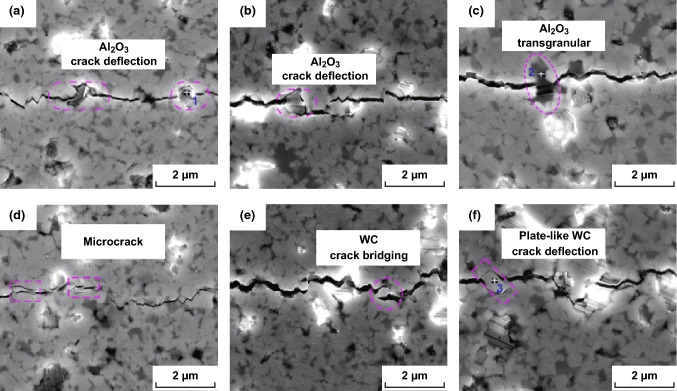
Toughening mechanisms in WC–Al_2_O_3_–TiC ceramics: **a, b** Al_2_O_3_ crack deflection, **c** Al_2_O_3_ transgranular, **d** microcracking, **e** WC crack bridging and **f** plate-like WC crack deflection [138]. Figure panels reproduced from Ref. [138] Elsevier copyright 2018

**Fig. 11:**
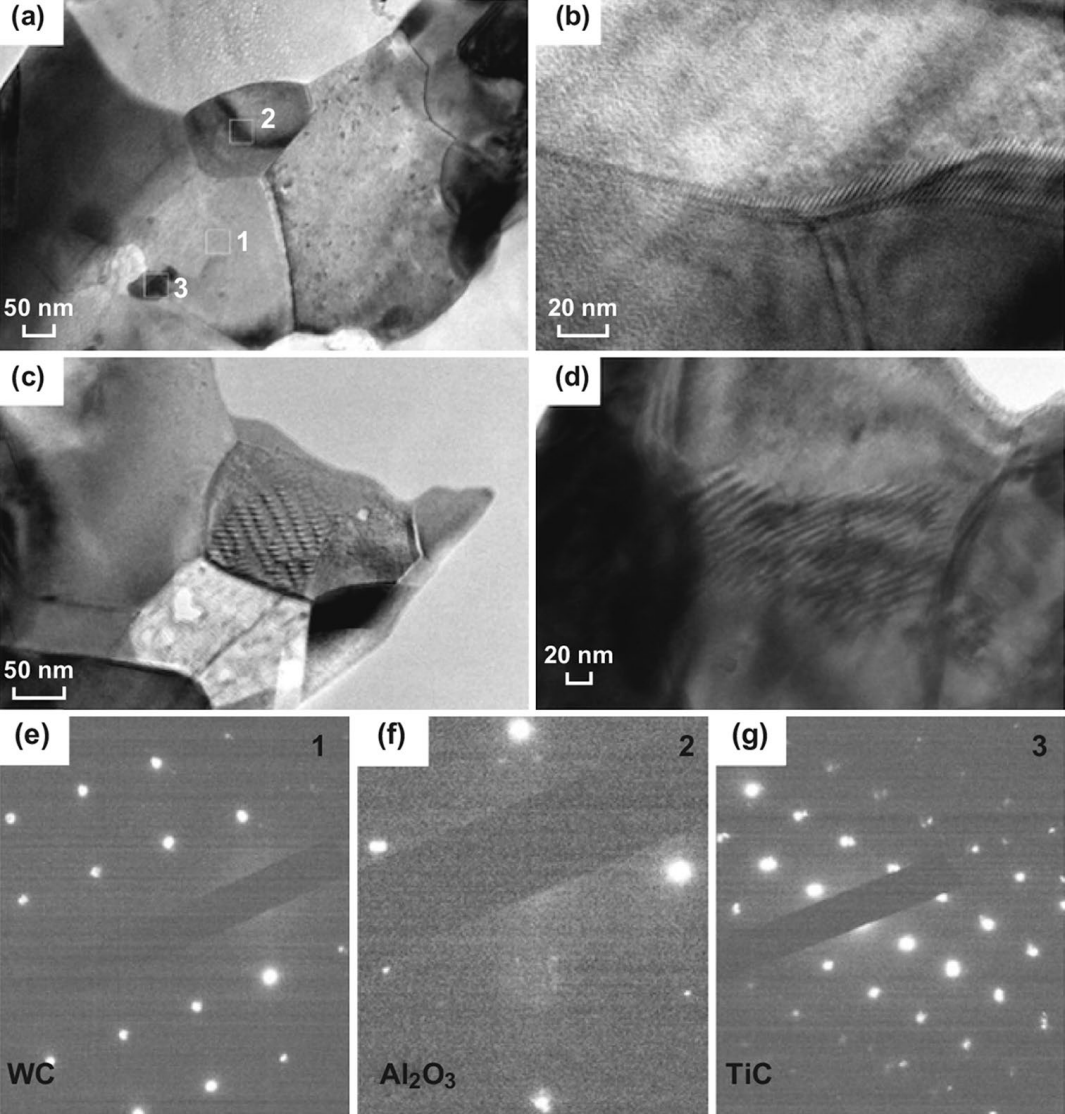
**a** TEM micrographs of WC–TiC–Al_2_O_3_ ceramics, **b-d** dislocation morphology, and electron diffraction spots of **e** point 1, **f** point 2, and **g** point 3 [138]. Figure panels reproduced from Ref. [138] with permission from Elsevier copyright 2018

Recently, we prepared nano-laminated WC–Al_2_O_3_–TiC ceramics with a high fracture toughness of 11.49 MPa m^1/2^ [138, 139]. Al_2_O_3_ crack deflection, transgranular Al_2_O_3_, microcracking, WC crack bridging, and plate-like WC crack deflection were the major toughening mechanisms (Fig. 10). Resulting from the thermal expansion mismatch between the secondary phase and the matrix, the residual stresses acting on WC/Al_2_O_3_ interfaces played a crucial role in the enhancement of grain boundary strength and then significantly improved the toughness of the matrix. As shown in Fig. 11, nanosized Al_2_O_3_ grains were located at the interface of WC grains and formed inter-granular structure, which hindered the WC grain growth and changed the grain shape. Nano-grained TiC grains were found to be embedded either in the WC matrix or at the WC grain boundaries, forming either intra-granular or inter-granular structure. Microcracks were found to be segregated inside alumina crystals (Fig. 11a). This might be a pinning effect caused by alumina grains and would substantially increase the number of potential sources of crack branching. Furthermore, dislocation morphologies were found to exist both at the WC/Al_2_O_3_ interfaces (Fig. 11b) and inside alumina crystals (Fig. 11c, d), which could increase the flaw tolerance of the material and enhance the toughness of the material.

### Transformation Toughening

Transformation toughening has been proved to be a significant approach to improve the toughness of ceramics [140–142]. Transformation toughening takes place, resulting from a stress-induced phase transformation of particulate inclusions in a ceramic matrix during fracture process, in which the most used particle is ZrO_2_ partially stabilized with Y_2_O_3_ or MgO. It depends on the transformation of metastable retained tetragonal zirconia to the stable monoclinic zirconia phase in the tensile stress field around a crack tip. Compressive stresses are introduced in the matrix due to shear deformation (1–7%) coupled with volume expansion (4–5%) resulting from monoclinic phase occupying more volume in comparison with tetragonal phase. The compresses, acting as closure stresses, reduce the local crack tip stress intensity, thereby enhancing the toughness of the matrix [143].

Significant toughness improvement was obtained for WC–ZrO_2_ composite by incorporating ZrO_2_ particles into WC matrix [109–111]. Mukhopadhyay et al. [144] reported that WC–6 wt% ZrO_2_ exhibited a toughness of 10.9 MPa m^1/2^, which is only modestly lower by 16% in comparison with that of reference WC–6 wt% Co cemented carbide, while the flexure strength enhanced by 18%. By comparing the XRD patterns of polished and fractured surface of same specimen, the author [104] found the presence of a strong m-ZrO_2_ peak with an intensity-reduced t-ZrO_2_ peak in the fractured surface, identifying the transformation of *t* → *m*-ZrO_2_ during the fracture process, which was the principle source of toughening. Furthermore, the author proposed that the toughness of WC–ZrO_2_ composite can be no more elevated with more than ~ 6 wt% ZrO_2_ addition. The presence of ZrO_2_ grains in the WC matrix as a discrete second phase enables the former to behave in an intrinsic manner, that is, to undergo the *t*–*m* transformation or to be retained as the metastable tetragonal form during cooling from the sintering temperatures. Thermally induced monoclinic transformation during the cooling process would result in the formation of microcracks around the transformed monoclinic ZrO_2_ particles. The microcracks are effective to toughen the materials by absorbing fracture energy, but the linkup or coalescence of the microcracks, as a result of added ZrO_2_ increasing, can reduce the toughness significantly. Therefore, it can be concluded that the highest toughness for WC–ZrO_2_ composite can be achieved by adding about ~ 6 wt% ZrO_2_ containing both tetragonal and monoclinic particles.

Wang [145] demonstrated that there existed a critical grain size for tetragonal ZrO_2_ retention during cooling from the sintering temperature to a given temperature, above which the tetragonal ZrO_2_ would transform to the monoclinic phase during the cooling process. In other words, the fraction of retained *t*-ZrO_2_ decreases with the increasing ZrO_2_ particle size. Therefore, a grain growth inhibitor addition is beneficial for the transformation toughening. Zheng et al. [146] reported that the toughness of WC–8 wt% ZrO_2_ was insensitive to the coarseness of microstructure and enhanced to 11 MPa m^1/2^ as a function of VC together with Cr_3_C_2_ addition.

### Whiskers Toughening

Profound toughening has been achieved in numbers of ceramics by incorporating whiskers. Compared with other toughening methods such as ZrO_2_ transformation toughening, whisker toughening enjoys a potential advantage that is the retention of high-toughness values at temperatures exceeding 1000 °C. Though sharing the similar toughness level with continuous-fiber-reinforced ceramics, whisker-reinforced composites possess advantages of easier fabrication as well as a higher degree of isotropy in mechanical properties because of the smaller aspect ratios involved. It is reported that five toughening mechanisms may be operative in whisker-toughened ceramics, that is, crack deflection, crack bowing, microcracking, whisker pullout, and crack bridging [147–149].

Though the number of researches and publications in whisker-toughened ceramics increased significantly, there are only a few reports with respect to BTC toughened by whiskers. Silicon carbide whiskers (SiC_w_), as a reinforcing agent, exhibited a prominent effect on improving the toughness of ceramics [150]. Chao et al. [151] suggested that it was feasible to toughen WC ceramics by using SiC_w_ instead of metallic binder phase. The toughness of WC–SiC_w_ was increased by 30-40% in comparison with pure WC, due to SiC_w_ addition. Sugiyama et al. [88, 152] reported that a highly tough WC–3 vol% SiC_w_ composites with an initial powder size of 0.71 μm, which were fabricated by SPS, had an improved fracture toughness about 7.8 MPa m^1/2^ in comparison with pure WC (about 5.9 MPa m^1/2^). The best toughening effect was achieved at 3–5 vol% SiC_w_ addition. Above 10 vol%, the toughness decreased from the maximums; however, it still maintained high levels above 7 MPa m^1/2^. Additionally, the author demonstrated that proper sintering parameter was of great importance to achieve the best toughening effect for the reason that an excessively high sintering temperature would made SiC_w_ thick, coarsen, and balloon and thus lowered the aspect ratio of the whiskers. Furthermore, Bengisu et al. [147] proposed that the amount of toughening would decrease significantly if the surface oxygen content of SiC_w_ was high. A possible solution to this problem is to eliminate the oxide formation on SiC_w_ surface by acid leaching cleaning the whiskers or coating the whiskers with an oxidation-resistant material.

**Fig. 12:**
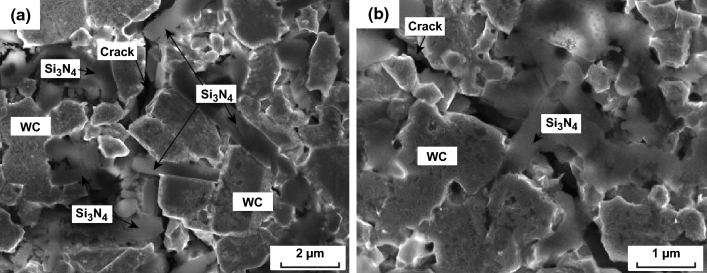
SEM images of indentation cracks observed in the sintered WC–Si_3_N_4_ composites: **a** Si_3_N_4_ whiskers pullout, **b** crack-bridging by Si_3_N_4_ whiskers [153]. Figure panels reproduced from Ref. [153] with permission from Elsevier copyright 2013

Besides SiC whisker, Si_3_N_4_ whisker is another most common ceramic whisker used as a toughening agent for ceramics. Zheng et al. [153–155] investigated the effect of in situ growing β-Si_3_N_4_ whiskers on toughening WC ceramics prepared by SPS. The formation of β-Si_3_N_4_ whiskers significantly improved the toughness to 10.94 MPa m^1/2^ for WC–Si_3_N_4_ sample from 6.69 MPa m^1/2^ for pure WC. The major toughening mechanisms were found to be Si_3_N_4w_ pullout and crack bridging by Si_3_N_4_ whiskers as shown in Fig. 12. Furthermore, the author found that at temperature above 1700 °C, β-Si_3_N_4_ grains ripened rapidly and grew into β-Si_3_N_4_ whiskers speedily; at 1450–1600 °C, WC grains grew slowly, while the growth of β-Si_3_N_4_ grain and the formation of β-Si_3_N_4_ whiskers were slow. Exploiting the kinetics difference between β-Si_3_N_4_ grain growth and WC grain growth, the author proposed a two-step sintering method. For sample heated to 1700 °C and then immediately cooled to 1600 °C with a holding time of 30 min, a plenty of Si_3_N_4_ whiskers formed and WC grains did not grow rapidly.

From an oxidation resistance point of view, Al_2_O_3_ whisker seems to be a more reasonable choice. Dong et al. [94] successfully fabricated WC–Al_2_O_3_ whiskers composites employing hot-pressing sintering as the assistance of VC as grain growth inhibitor. Maximum toughness (13.8 MPa m^1/2^) was obtained for WC–10 wt% Al_2_O_3w_ composites. The toughening mechanisms were crack bridging, crack deflection as well as ligamentary bridging between crack surfaces. When the whisker content was above 10 wt%, the toughness decreased with the increasing whiskers content. Generally speaking, whisker addition deteriorates the densification process, due to whiskers resisting particle rearrangement, resulting from extensive sliding distances along whisker boundaries during sintering as well as at high whisker aspect ratios, or ratios above a critical volume fraction [147]. Therefore, there are two opposing factors operating in whisker-reinforced composites, and those are toughening effect and densification deteriorating influence. Densification becomes more difficult with the increasing whisker content, resulting from the formation of a rigid network as a result of whiskers coming into contact with each other. As the whiskers further increased beyond a critical value, for example 10 wt% in Dong’s research [94], the densification deteriorating influence started dominating and then the toughness was decreased.

### Laminated Structures and Functional Gradient Materials (FGM)

Besides optimizing the chemical composition, another strategy to enhance the fracture toughness of ceramics is through the design of ceramic laminates. Residual stresses are inevitably set up during the cooling of layered ceramics, due to the mismatch in thermal expansion coefficients as well as elastic moduli. Lakshminarayanan et al. [156] demonstrated that a residual compression stress of about 400 MPa developed in the surface layer of a three-layered Al_2_O_3_–ZrO_2_ ceramics significantly enhanced the fracture toughness to 30 MPa m^1/2^ where the intrinsic fracture toughness was only 5–7 MPa m^1/2^. Blugan et al. [157, 158] reported that apparent fracture toughness of 18 MPa m^1/2^ was obtained for micro-laminated Si_3_N_4_–TiN composites, as a result of the formation of surface compressive stresses. It can be concluded that the residual stresses can be tailored to obtain high surface compressive stress, resulting in the great enhancement of fracture toughness.

**Fig. 13:**
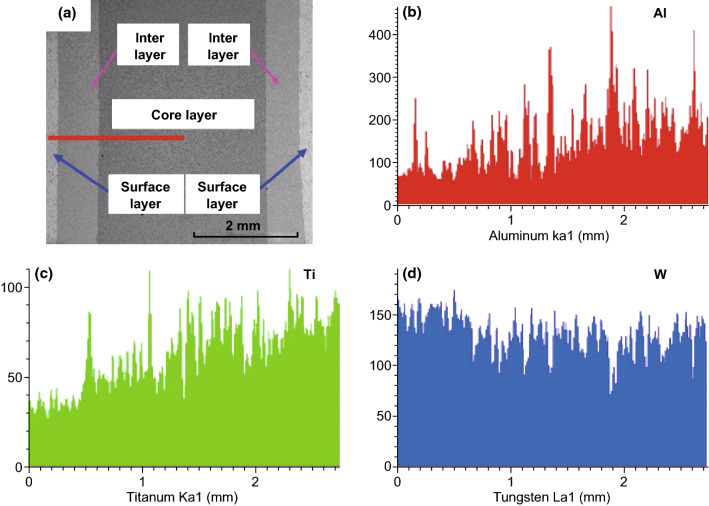
**a** SEM micrographs and EDS maps of the distribution of **b** Al **c** Ti, and **d** W elements on the red line of nano-laminated WC–Al_2_O_3_–TiC ceramics [139]. Figure panels reproduced from Ref. [139] with permission from Elsevier copyright 2017. (Color figure online)

Recently, we successfully prepared nano-laminated WC–Al_2_O_3_–TiC composites with the assistance of Cr_3_C_2_ and VC as grain growth inhibitor (Fig. 13) [139]. Because the coefficient of thermal expansion of TiC (7.74 × 10^−6^/°C) and Al_2_O_3_ (8.8 × 10^−6^/°C) is higher than that of WC (3.84 × 10^−6^/°C), an increase content of TiC and Al_2_O_3_ from surface layer to core layer and a decrease content of WC from surface layer to core layer contributed to the generation of significant residual compressive stress on the surface of material, leading to the significant toughness enhancement.

### New-Concept Toughening (Carbon Nanotube and Graphene Toughening)

Carbon nanotube (CNT), a one-dimensional (1D) nano-material discovered by Iijima [159] in 1991, has captured tremendous interests among scientific community and industry, owing to its high aspect ratio together with extraordinary mechanical, electronic, thermal, magnetic, and optical characteristics [160]. Many of these exceptional properties can be effectively exploited through incorporating carbon nanotube into BTC matrix, aiming to significantly improve the mechanical properties, fracture behavior, and functional features.

**Fig. 14:**
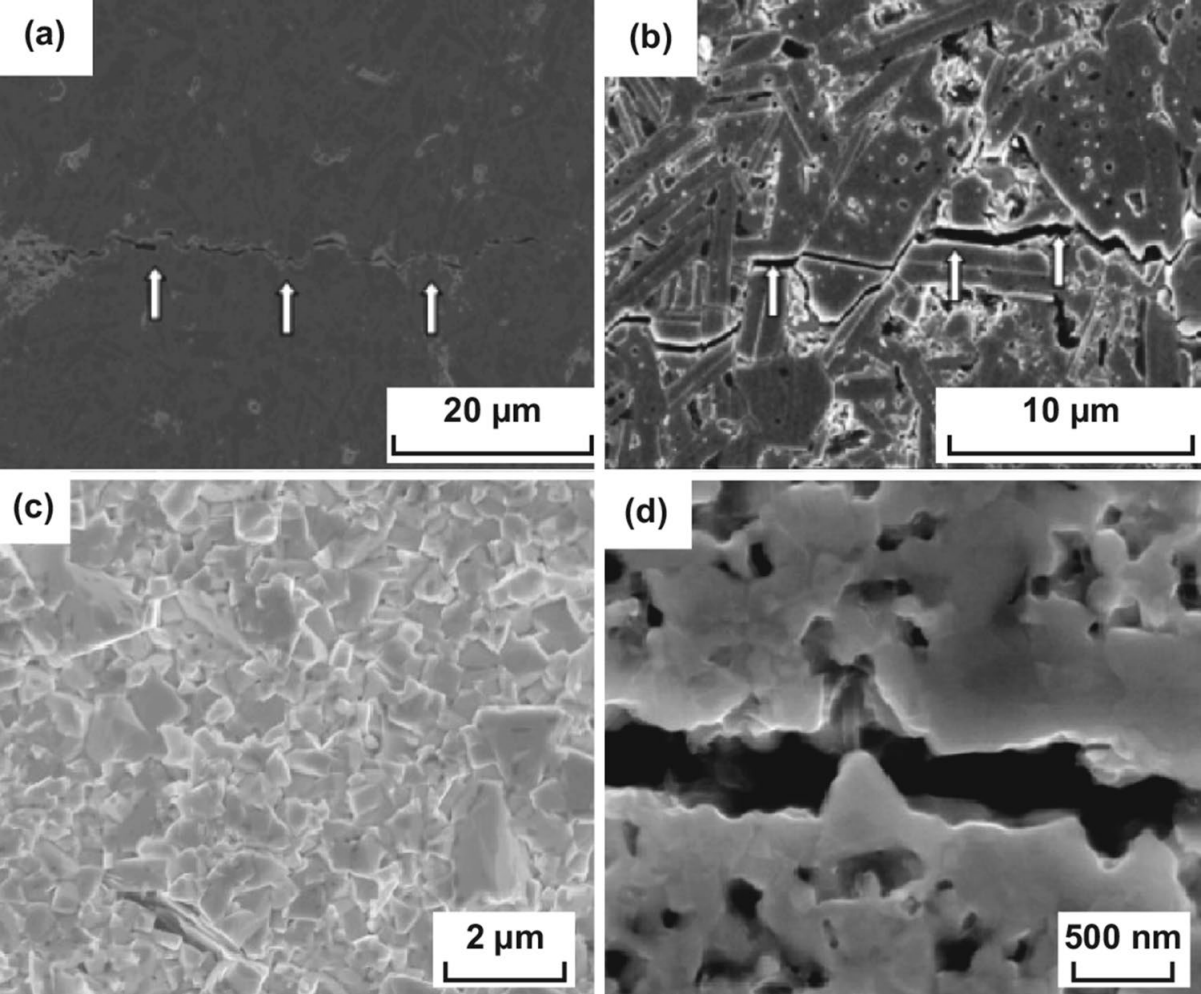
FE-SEM micrographs of crack propagation in **a** WC–5 vol% CNT and **b** WC–10 vol% CNT [161]. Typical SEM images of **c** the fracture surfaces and **d** crack details of WC–CNT composites [166]. Figure panels reproduced from Ref. [161] with permission from INST Problems Mechanical Engineering-Russian Card Sciences copyright 2011, Ref. [166] with permission from Elsevier copyright 2018

Shon et al. [161] reported the production of highly dense WC–CNT composites employing HFIHS under a pressure of 80 MPa within 3 min. The fracture toughness of obtained WC, WC–5 vol% CNT, and WC–10 vol% CNT was 7, 10.5, and 11 MPa m^1/2^, suggesting that CNT addition significantly enhanced the toughness of BTC. The primary toughening mechanism was crack deflection by CNT as shown in Fig. 14a, b. Furthermore, the author demonstrated that the CNT addition was beneficial for the densification of WC. Bai et al. [162–164] demonstrated that adding CNT into WC-based matrix significantly enhanced the toughness, resulting from crack bridging, CNTs debonding, crack deflection, and CNT pullout. Jang et al. [165] reported that 46.8% increment in fracture toughness was obtained for WC as a function of 15 vol% CNT addition, demonstrating that CNT prevented crack propagation by shielding a stress field in front of the crack tip or by bridging the crack forming ligaments behind the crack tip. Cao et al. [166] prepared ultra-fine-grained WC–1.0 wt% CNT composites with a high toughness of 9.11 MPa m^1/2^ using SPS and investigated the effect of sintering temperature on the structure of CNT. In addition to crack deflection by CNT, CNT pullout was identified as another toughening mechanism as illustrated in Fig. 14c, d. Moreover, the author found that the structure of CNT was destroyed and CNT transformed to graphite phase in 1900 °C, indicating that WC–CNT composites should be sintered at moderate temperatures.

Besides CNT, graphene is another nanocarbon material, but possesses a unique two-dimensional (2D) *sp*^2^ structure with considerable theoretical specific surface area (~ 2630 m^2^ g^−1^) and distinctive mechanical properties (e.g., breaking strength 42 N m^−1^, tensile strength 130 GPa, stiffness 300–400 N m^−1^, thermal conductivity 3000–5000 W m^−1^ K^−1^, spring constant 1–5 N m^−1^, and Young’s modulus 0.5–1 TPa) [167, 168]. The 2D structure endows graphene with inherent advantages over other carbon allotropes as CNT. Furthermore, graphene possesses better dispersion properties than CNT and can be homogeneously dispersed into a ceramic matrix, which is another rather advantageous factor in improving the microstructure and mechanical properties of the ceramic composites [169, 170]. Additionally, graphene is endowed with energy dissipating mechanism by generating graphene bending and kinking. The coordination deformation of graphene consumes strain energy and then reduces the crack extension energy, significantly contributing to the improvement of fracture toughness [171]. All these properties are very attractive for the application of graphene as reinforcement for ceramic composites [172].

It is reported that graphene showed remarkable effectiveness in enhancing the fracture toughness of Al_2_O_3_-based ceramics [173], Si_3_N_4_-based ceramics [174], ZrB_2_-based ceramics [175], ZrO_2_-based ceramics [176], and TaC-based ceramics [177]. To the best of our knowledge, few studies have been reported on the effects of graphene addition on the densification and properties of BTC materials hitherto.

**Fig. 15:**
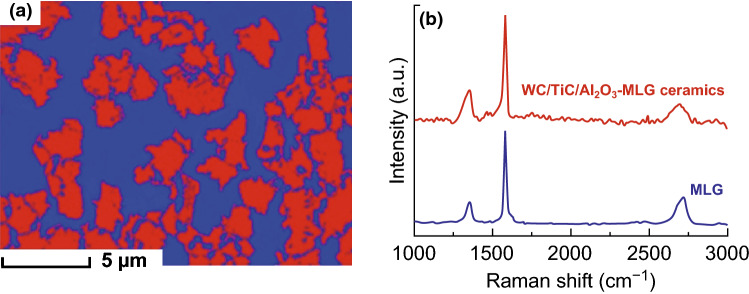
**a** The distribution of MLG (color in red) in WC-based composites reinforced by 0.1 wt% MLG (by EBSD). **b** Raman spectroscopy of MLG and sintered ceramics [178]. Figure panels reproduced from Ref. [178] with permission from Elsevier copyright 2017. (Color figure online)

**Fig. 16:**
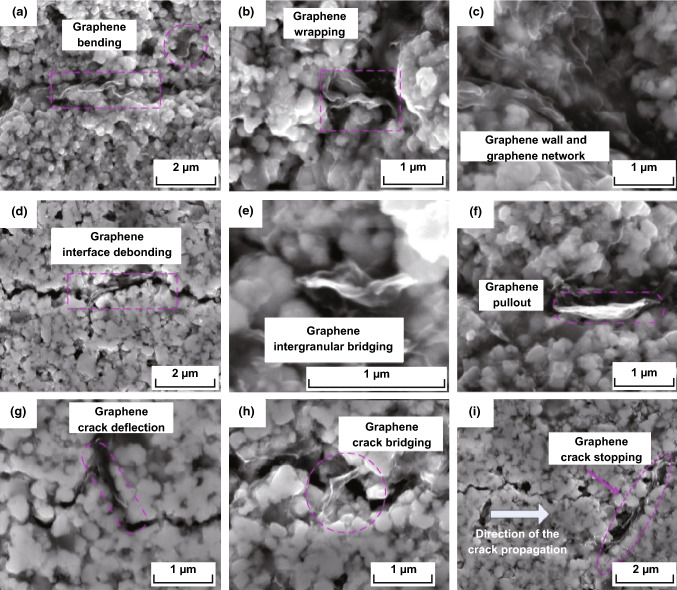
Toughening mechanisms in WC-based ceramics reinforced by MLG: **a** graphene bending, **b** graphene wrapping, **c** graphene wall and network, **d** graphene interface debonding, **e** graphene intergranular bridging, **f** graphene pullout, **g** graphene deflection, **h** crack bridging, and **i** crack stopping [178]. Figure panels reproduced from Ref. [178] with permission from Elsevier copyright 2017

Recently, we investigated the toughening effect of multilayer graphene (MLG) on BTC through hot-pressing method [178]. It is worthwhile mentioning that the achievement of good graphene/WC composites depends extremely on three critical factors, and those are homogeneous dispersion within the WC matrix, minimization of the mechanical damage of graphene, and optimum bonding between WC matrix and graphene. MLG was homogeneously dispersed in the WC-based ceramic matrix as shown in Fig. 15a. Figure 15b depicts Raman spectroscopy of MLG and sintered ceramics, implying the survival of MLG after the so-designed two-step sintering. Both the spectra presented the characteristic D, G, and 2D bands corresponding to MLG at ∼ 1350, ∼ 1580, and ∼ 2700 cm^−1^, confirming that the graphene-based structure has not been damaged during the milling and sintering processes. WC-based ceramics with 0.1 wt% MLG addition exhibited a fracture toughness of 14.1 MPa m^1/2^, undergoing ~ 53.3% enhancement in comparison with monolithic ceramics. MLG bending, wrapping, interface debonding, MLG wall and network, MLG-induced weak interface, grains bridging by MLG, MLG pullout, crack deflection, crack bridging, and crack stopping were the major toughening mechanisms (Fig. 16). Also, the preferential orientation of MLG in WC matrix and MLG intrinsic energy dissipating mechanisms as sheet kinking, bending, and sliding played very significant roles in contributing to the toughness enhancement of BTC reinforced by MLG. The detailed discussion about the toughening mechanism in WC/MLG composites can be found in our previous article [178].

### Synergistic Toughening

An area of remarkable potential is the synergistic toughening, taking advantage of the possibilities of multiple toughening mechanisms operative within a single composite. It is suggested from both theoretical analysis and experimental observations that the overall effect of combined toughening mechanisms may be greater than the sum of the increments from individual components.

Combining secondary-phase reinforcements, such as platelets as well as whiskers, with microstructural tailoring of the matrix may be of great potential. Also, transformation toughening could be combined with other toughening methods. Furthermore, toughening by designing laminated structure combined with toughening by the design of chemical composition would be of significant promise to improve the fracture toughness of ceramics. By combining particle dispersion toughening (with Al_2_O_3_) with transformation toughening (with ZrO_2_), Xia et al. [128] prepared WC–Al_2_O_3_–ZrO_2_ composites with high fracture toughness. Bai et al. [163] reported a highly tough WC–Al_2_O_3_–CNT composite coupling carbon nanotube toughening with Al_2_O_3_ particle dispersion toughening. Combining particle dispersion toughening, graphene toughening, and laminated structure toughening, we prepared highly tough laminated WC–Al_2_O_3_–TiC–MLG composite [178].

## Mechanical Properties of Binderless Tungsten Carbide

Mechanical properties of BTC are strongly dependent on their densification degree, grain size, chemical compositions as well as microstructure. Hardness and fracture toughness, the critical properties in many applications of engineering structural materials, are the most important intrinsic mechanical properties for BTC. Despite its high hardness as well as excellent wear/corrosion/oxidation resistance, a major limitation of BTC’s further applications, structural application as an example, is its poor fracture toughness. Enhancing the fracture toughness without sacrificing hardness is invariably one of the goals of BTC development. Wear resistance of BTC is also one of the most significant mechanical properties; however, it is mostly determined by the hardness coupled with fracture toughness.

### Hardness Versus Fracture Toughness

Hardness is always a stress standing for the resistance to non-recoverable deformation of a material, while fracture toughness characterizes a material’s resistance to crack propagation and, as such, is measured as the fracture energy. They are the two basic mechanical properties of BTC and the foundation of other mechanical properties, such as wear resistance, flexural strength as well as impact resistance.

**Fig. 17:**
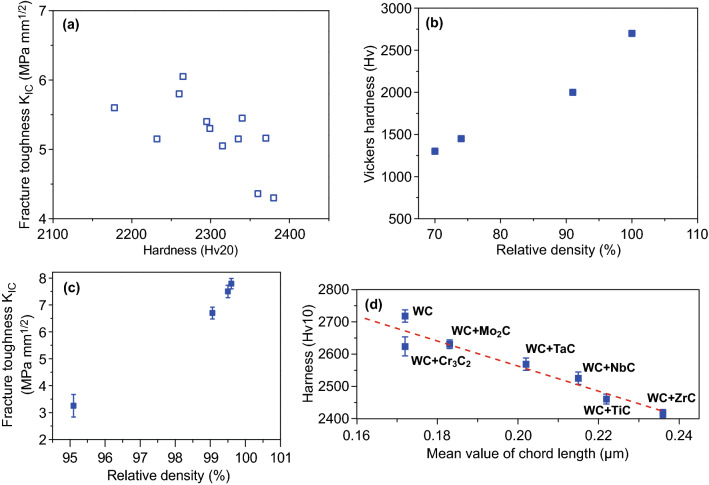
**a** Plot of fracture toughness versus hardness of BTC [126]. **b** Plot of hardness and **c** fracture toughness versus relative density [119, 179]. **d** Plot of hardness versus chord length of dense samples [180]. Figure panels reproduced from Ref. [126] with permission from IEEE copyright 2005, Ref. [119] with permission from Elsevier copyright 2006, Ref. [179] with permission from Elsevier copyright 2017, Ref. [180] with permission from EPMA copyright 2014

The mechanical properties of pure WC were reported in several researches. Date demonstrates that the fracture toughness of pure WC decreased with the increasing hardness (Fig. 17a), which is similar to the general tendency of conventional WC–Co cemented carbide. It should be noted that for WC–Co cemented carbides, Co binder phase acts for impeding the crack propagation through shielding a stress in front of crack tip. However, in pure WC, crack propagates along the WC/WC interface or penetrates into large WC grains, implying that the large WC grains together with the remaining small pores prevent crack growth and absorb fracture energy. Furthermore, the mechanical properties of BTC can be remarkably enhanced by improving the densification. As shown in Fig. 17b, c, both hardness and fracture toughness were improved with the increase in the relative density, and high mechanical properties (hardness and fracture toughness) corresponded to high density. Also, it is revealed that Hall–Petch-like relationship is applied to BTC as shown in Fig. 17d, implying that hardness enhancement of BTC is associated primarily with obtaining fine grain in the sintered material at a given relative density.

It has been recognized that the reduction in microstructural scale to nanometer resulted in substantial enhancement in mechanical properties of BTC. The trade-off relationship between hardness and fracture toughness may be reduced when the grain sizes approach nanoscale as 100 nm. With respect to conventional BTC, the fracture toughness reduces with increasing hardness; however, the hardness increase in nanosized BTC does not lower or even increase the fracture toughness. Though possessing higher relative density (99.1%), micro-sized WC (400 nm) exhibited both lower hardness (27.5 GPa) and toughness (4.5 MPa m^1/2^) in comparison with nanosized WC (87 nm) having a hardness of 29.6 GPa and toughness of 7.1 MPa m^1/2^ [130, 181].

Table 6 illustrates hardness and fracture toughness of some reported BTC with various chemical compositions, relative densities, and grain sizes. As discussed before, some carbide addition can improve the sinterability of BTC; however, the carbide-added BTC, such as WC–TiC, WC–VC, and WC–TaC, exhibited a deteriorated hardness and similar or slightly lower fracture toughness in comparison with pure WC. Metal-oxide-added WC-based composites, such as WC–Al_2_O_3_, WC–MgO, and WC–ZrO_2_, showed a good combined property similar to or even better than conventional WC–Co cemented carbide. Grain growth inhibitor is essential for WC-based composites to further improve mechanical properties, regardless of carbide addition or oxide addition. Graphene-reinforced WC-based composites exhibited the best combined property.

**Table 6:** Hardness and fracture toughness of some reported BTCs

Material	Relative density (%)	Sintered grain size (nm)	Hardness^a^ (Hv)	Fracture toughness^b^ (MPa m^1/2^)
WC [18]	99.2	200	2925	8.9
WC [181]	100	130	3061	7.3
WC [182]	98.5	380	2854	7.1
WC [130]	99	87	3020	7.1
WC [125]	99.8	220	2959	7.2
WC [58]	97.6	360	2480	6.6
WC–20 at.% TiC [81]	98.5	200	2032	6.3
WC–20 at.% TiC [183]	99	200	2240	7.5
WC–3 wt% TiC–2 wt% TaC–0.2 wt% Cr_3_C_2_–0.2 wt% VC [184]		600	2300	7.9
WC–6 wt% Mo_2_C [184]		250	2400	8.4
WC [84]	99.9	280	2795	4.38
WC–1 wt% VC [84]	96.5	280	2795	4.2
WC–1 wt% VC [185]	99.8	272	2585	6.9
WC–1 wt% Cr_3_C_2_ [185]	100	277	2605	7.2
WC–0.3 wt% VC–0.5 wt% Cr_3_C_2_ [119]	97.5	240	2875^d^	6.05
WC–0.33 wt% VC–0.54 wt% Cr_3_C_2_ [121]	97.9	730	2464	4.4
WC [180]	100.6	171	2720	7.0
WC–1 wt% Mo_2_C [180]	100.8	183	2630	6.6
WC–1 wt% Mo_2_C [85]	99	450	2461	4.8
WC–1 wt% TaC [180]	99.7	202	2570	6.9
WC–1 wt% ZrC [180]	98.8	236	2420	6.5
WC–1 wt% NbC [180]	99.6	214	2540	6.6
WC–6 mol% SiC–2 mol% ZrC [71]	99	720	2193^e^	6.7
WC–20 mol% SiC–0.3 mol% Cr_3_C_2_ [90]	99	420	2193	6.4
(WC–0.8 mol%Cr_3_C_2_)–5 vol% SiC_w_ [152]	100.4	1500	2041	7
WC–14.3 wt% Al_2_O_3_–0.5 wt% VC [93]	98	2000	2103	11.54
WC–10 wt% Al_2_O_3w_–0.5 wt% VC [94]	98	2000	2103	13.8
WC–10 vol% Al_2_O_3_ [102]	99.8	101	2540	9.4
WC–4.3 wt% MgO [135]	99	2590	1878^f^	12.95
WC–6 wt% ZrO_2_ [104]	100	660	1876	10.8
WC–3 wt% AlN [186]	99.6	700	2400	7.5
WC–10 wt% Si_3_N_4w_ [153]	100	1260	1801	10.94
WC–1.0 wt% CNT [166]	101	200	2328	8.95
WC–3 wt% Al_2_O_3_–2 wt% TiC–0.15 wt% MLG^c^ [178]	98.9	400	2255	14.5

^a^For convenience of comparison, the original hardness values from papers were converted to Vickers hardness values

^b^Fracture toughness value was calculated from indentation method

^c^This is the surface layer of a functionally graded WC–AL_2_O_3_–TiC–MLG composites

^d^This is a value of HV0.5

^e^This is a value of HV1.0

^f^This is a value of HV30

### Wear Resistance

Liu et al. [187] studied the wear resistance of pure WC. The experiments were ball-on-disk type, in which ball is the SiC counterpart ball with a diameter of 6.25 mm and a hardness of 28 GPa, and disk is WC. The test temperature increased from 25 to 800 °C under air and vacuum conditions. The results showed that pure WC can maintain phase stability at the temperature till 800 °C in vacuum. In air, the wear mechanism of pure WC was mainly oxidation wear, and the critical temperatures causing degradation on the wear properties were 500 and 550 °C.

**Fig. 18:**
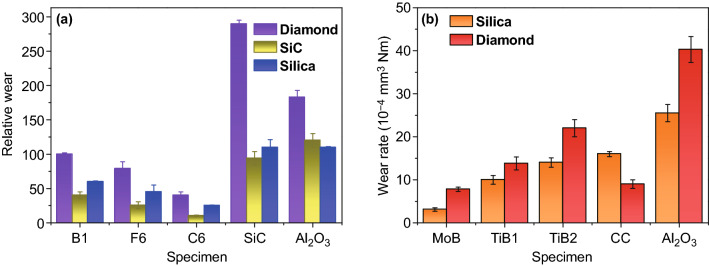
**a** Relative wear rates for the tested materials as shown in Table 7 abraded with diamond, SiC, and silica. 100 corresponds to Bl worn with diamond [188]. **b** Wear rates for the different tested materials as shown in Table 8 abraded with silica and diamond [184]. Figure panels reproduced from Ref. [188] with permission from Springer Nature 1998, Ref. [184] with permission from Wiley copyright 2000

Engqvist et al. [188] compared the sliding wear resistance of WC–TiC–TaC with conventional WC–Co cemented carbides and ceramics as Al_2_O_3_ and SiC (Table 7). Three kinds of abrasive particles, including diamond, silicon carbide, and silica, were used. During the test, the normal load was 0.2 N and the total sliding distance was 50 m. The results showed that the WC–TiC–TaC had a slightly lower wear resistance than WC–Co cemented carbides, but much higher than that of common engineering ceramics Al_2_O_3_ and SiC as demonstrated in Fig. 18a. The BTC was worn by a preferential removal of TiC grains, leaving the TaC/NbC and WC grains unsupported, which contributed to an increased wear rate in comparison with WC–Co cemented carbides, implying that TiC was a weak constituent of the binderless carbide.

**Table 7:** Composition, grain size, hardness, and fracture toughness of the tested materials [188]

Specimen	Composition (wt%)	Grain size (μm)	Hardness (HV0.5)	Fracture toughness K_IC_ (MPa m^1/2^)
B1	94.5WC, 2Ta/NbC, 3TiC, < 0.5Co	1.3	1900	7.9
F6	94WC, 5.4Co, 0.6(Ta, Nb)C	1.0	1720	10.8
C6	94WC, 6Co	7.0	1220	15
SiC	SiC + some free C	3.0	2300	4.5
Al_2_O_3_	96.95Al_2_O_3_, 0.25MgO, 2.8ZrO_2_	1.9	1820	5.4

Therefore, it is denoted that BTC composites free of TiC may have a superior wear resistance to conventional WC–Co cemented carbides. Based on this idea, Botton et al. [184] investigated the wear resistance of WC–6 wt% Mo_2_C and WC–6 wt% Co (Table 8) as shown in Fig. 18b. The results demonstrated that WC–Mo_2_C exhibited better wear resistance than WC–Co cemented carbides. The wear mechanism of WC–Mo_2_C was found to be ductile, which was different from that of WC–TiC–TaC suffering from grain pullout of TiC. It seems that the wear resistance of BTC strongly depends on the bonding between the carbide grains, and WC–Mo_2_C provided a better bonding than WC–TiC–TaC.

**Table 8:** Composition, grain size, hardness and fracture toughness of the tested materials

Specimen	Composition (wt%)	Grain size (μm)	Hardness (HV0.5)	Fracture toughness K_IC_ (MPa m^1/2^)
MoB [184]	94WC, 6Mo_2_O	0.25	2400	8.4
TiB1 [184]	94.6WC, 3TiC, 2TaC, 0.2Cr_3_C_2_, 0.2VC	0.6	2300	7.9
TiB2 [188]	95WC, 3TiC, 2TaC	2.0	1900	7.9
CC [188]	94WC, 6Co	7	1200	15
Al_2_O_3_ [188]	96.95Al_2_O_3_, 0.25MgO, 2.8ZrO_2_	1.9	1820	5.4

Venkateswaran et al. [106, 107] investigated the wear resistance of WC–ZrO_2_ composites using a fretting wear tester employing bearing steel as a counter body on WC–ZrO_2_ under the ambient condition of temperature and humidity, demonstrating that the abrasion and tribochemical wear resulting in WO_3_ formation were the predominant wear mechanisms for WC–ZrO_2_ composites.

**Fig. 19:**
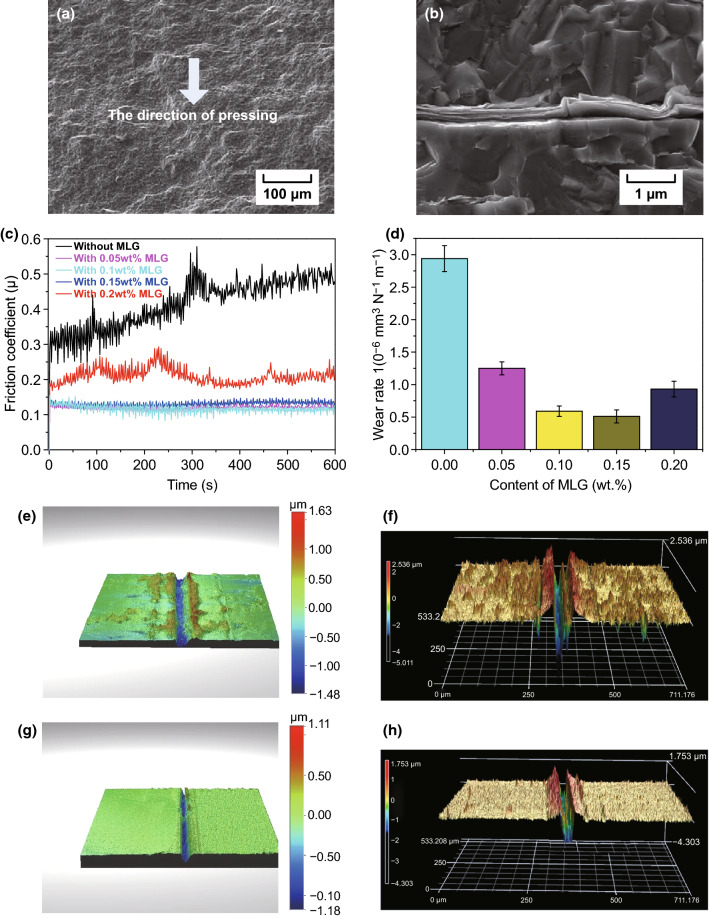
Preferential orientation of MLG in WC ceramics after hot-pressing **a** in low magnification and **b** in high magnification, influence of MLG contents on **c** friction coefficient and **d** wear rates, surface topographies of wear tracks on **e**, **f** monolithic WC ceramics and **g**, **h** ceramics containing 0.1 wt% MLG [178]. Figure panels reproduced from Ref. [178] with permission from Elsevier copyright 2017

**Fig. 20:**
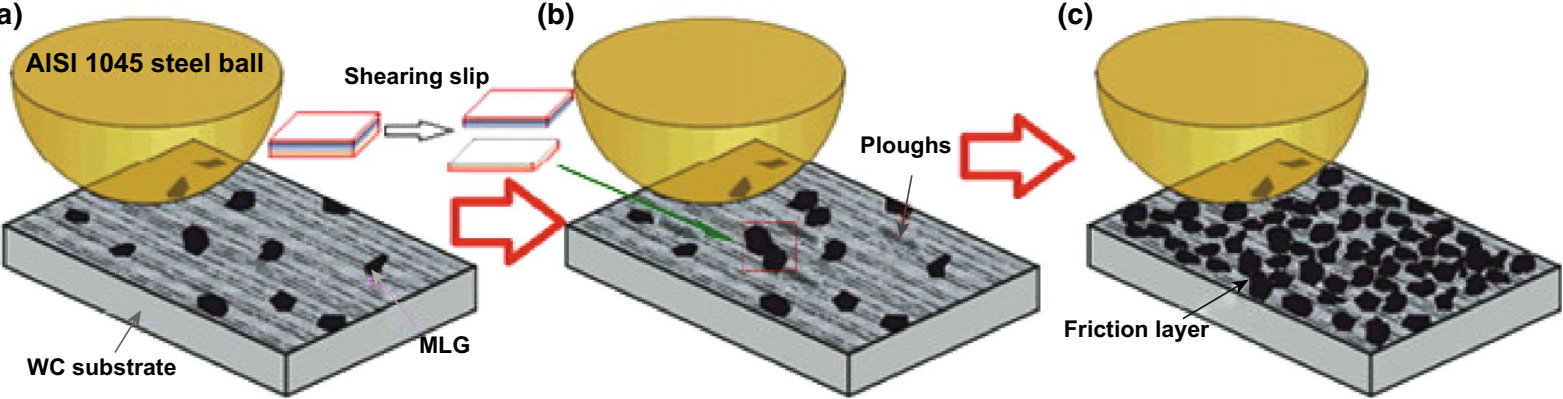
Schematic of effect mechanism of MLG reinforced WC-based materials against AISI 1045 steel [178]. Figure panels reproduced from Ref. [178] with permission from Elsevier copyright 2017

Recently, we investigated the sliding wear resistance of MLG-reinforced BTC [178]. The results showed that the ab plane of MLG exhibited a preferential orientation perpendicular to the direction of applied pressing as shown in Fig. 19a, b, playing a rather significant role in enhancing the tribological performance of MLG-reinforced materials. MLG-reinforced WC exhibited ~ 73.8% decrement in friction coefficient and ~ 82.65% improvement in wear resistance in surface perpendicular to the direction of applied pressing in comparison with monolithic ceramics as illustrated in Fig. 19c, d. It is noted from Fig. 19e–h that both the depth and the width of the wear track of WC ceramics containing 0.1 wt% MLG are smaller than those of monolithic ceramics. The dramatic improvement in tribological performance is attributed to the self-lubrication of MLG and easily formed friction layer in the contact interface as shown in Fig. 20. Furthermore, the unrivaled thermal conductivity of MLG and its rather significant effect in inhibiting the grain growth are an important contribution to the improved tribological performance.

## Concluding Remarks and Outlook

In this review, recent extensive investigations on the processing, microstructure, and mechanical properties of BTC and BTC composites are summarized. Such insights should be very helpful in tailoring the properties by judiciously selecting the chemical composition coupled with processing techniques and parameters.

Critical issue associated with the densification of BTC has been reviewed. It is believed that the densification of BTC is rather challenging due to the absence of metallic binder. The review highlights the crucial roles of mixed carbon content, carbide grain size, ceramic additive as well as advanced sintering techniques in the densification process. Carbon control is rather essential for BTC achieving maximum densification and performance levels. Strict design of additives including carbon, WO_3_, and W together with carefully control of thermal processing process is of great importance to the carbon content in final product. Sintering temperature strongly depends on the grain size; thus, finer initial powder grain size is helpful in lowering the sintering temperature as well as shortening the sintering process, especially for nanopowders. Nanocrystalline WC powders possess better sinterability in comparison with micro-sized ones, due to their high surface energy increasing the sintering driving forces. Besides reducing the particle size of WC matrix, it is also effective to facilitate the sintering of WC by dispersing some nanosized second-phase additive within micro-sized WC matrix grains or at the grain boundaries of WC matrix. The low melting point of the metal and possibility of carbonation and oxidation make the selection of the transition-metal carbides (TiC, TaC, and SiC) and metal oxides (Al_2_O_3_, ZrO_2_, Y_2_O_3_, and La_2_O_3_) binders more favorable for consolidating WC powders. The advanced sintering techniques, such as SPS, RSPS, HFIHS as well as PCAS, are successfully employed in laboratory scale preparing dense BTC. Particularly, SPS is currently the most commonly used sintering method to consolidate BTC or BTC composites.

Another critical issue associated with the toughening of BTC has also been reviewed. Varieties of traditional toughening methods including particle dispersion toughening (Al_2_O_3_, MgO, etc.), transformation toughening (ZrO_2_), whisker toughening (SiC_w_, Si_3_N_4w_, Al_2_O_3w_), and synergistic toughening together with some new-concept toughening methods, such as laminated structure toughening, carbon nanotube toughening, and graphene toughening, have been proposed to toughening BTC. Particularly, new-concept toughening methods offered great promise of a revolutionary advance in the production of highly tough BTC. Furthermore, toughening by designing laminated structure combined with toughening by the design of chemical composition would be of significant promise to enhance the fracture toughness of BTC.

Mechanical properties of BTC are strongly dependent on their densification degree, grain size, chemical compositions as well as microstructure. The fracture toughness of pure WC decreases with the increasing hardness, which is similar to the general tendency of conventional WC–Co. Both hardness and fracture toughness can be improved with the increase in the relative density, and high mechanical properties (hardness and fracture toughness) correspond to high densification. The trade-off relationship between hardness and fracture toughness may be reduced when the grain sizes approach nanoscale as 100 nm. Compared with other materials reinforced BTC, graphene-reinforced BTC exhibited the best combined property. BTC has comparable or higher wear resistance than conventional WC–Co cemented carbides, but much higher than that of common engineering ceramics such as Al_2_O_3_ and SiC.

Finally, it should be noted that, in spite of the progress made in the processing as well as properties of BTC, this remains a field of prospect, and further research is required to optimize chemical composition, further enhance mechanical properties as well as reduce manufacturing costs, develop sintering techniques for large scale production, develop new techniques as selective laser sintering for fabricating complex-shaped functional BTC composite powder parts, and advance them more widely and novel applications, so as to produce BTC or BTC composites on an industrial or commercial scale.
